# Precise Control of Charge–Structure Coupling in Polyamide Membranes for Mono‐ and Divalent Ion Separation

**DOI:** 10.1002/advs.77377

**Published:** 2026-08-25

**Authors:** Shaofan Duan, Shuai Jiang, Feidong Yang, Yuxuan Feng, Yongxuan Shi, Pengfei Zhang, Mengyang Hu, Yuqing Lin, Tae Hoon Lee, Kecheng Guan, Keizo Nakagawa, Tomohisa Yoshioka, Hideto Matsuyama, Eiji Kamio

**Affiliations:** ^1^ Research Center for Membrane and Film Technology Kobe University Nada Kobe Japan; ^2^ Department of Chemical Science and Engineering Kobe University Nada Kobe Japan; ^3^ Department of Future Energy Engineering Sungkyunkwan University Suwon Republic of Korea; ^4^ Graduate School of Science, Technology and Innovation Kobe University Nada Kobe Japan

**Keywords:** interfacial polymerization, mono/divalent ion separation, tunable compositions, zwitterionic copolymer grafting

## Abstract

Selective separation of monovalent and divalent ions sharing the same charge sign is critical for treating complex aqueous streams such as natural brines and industrial wastewater. However, conventional nanofiltration (NF) membranes, which rely primarily on charge‐based exclusion, often struggle to achieve efficient separation of counter‐ions, limiting their applicability. Herein, we report a composition‐regulated zwitterionic copolymer grafting strategy that enables precise tuning of membrane surface chemistry and pore structure of NF membranes. A zwitterionic copolymer, poly(2‐methacryloyloxyethyl phosphorylcholine‐co‐2‐aminoethyl methacrylate hydrochloride) (P(MPC‐co‐AEMA)), with tunable MPC/AEMA ratios, is grafted onto the polyamide (PA) selective layer via secondary interfacial polymerization. Rather than relying solely on the introduction of zwitterionic functionalities, this approach leverages the balance between reactive anchoring segments (AEMA) and zwitterionic segments (MPC) to regulate grafting density, surface charge, and structural evolution of the PA layer. Through systematic control of copolymer composition, the membrane properties can be finely tuned from strongly charged to near‐neutral surfaces with concurrently reduced pore size, thereby suppressing charge‐sign‐dependent electrostatic interaction effects and promoting sterically governed ion transport. This work demonstrates that balancing reactive anchoring and zwitterionic segments offers a rational strategy for tuning membrane physicochemical properties and advancing NF membranes toward charge‐sign‐independent ion‐selective separations.

## Graphical abstract

A zwitterionic copolymer, Poly(2‐methacryloyloxyethyl phosphorylcholine‐co‐2‐aminoethyl methacrylate hydrochloride) (P(MPC‐co‐AEMA)) copolymers, with tunable MPC/AEMA ratios, is grafted onto the polyamide (PA) selective layer via secondary interfacial polymerization. Balancing reactive anchoring segments (AEMA) and zwitterionic segments (MPC) regulated grafting density, surface charge, and PA structure, producing a near‐neutral membrane surface with controlled pore size, thereby enabling charge‐sign‐independent monovalent/divalent ion separation.

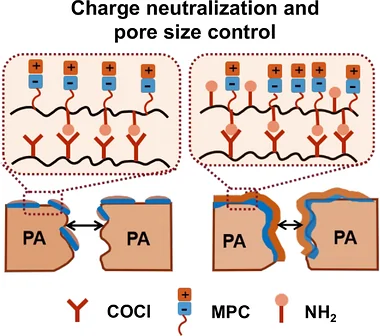

## 1 Introduction

Recovering valuable resources from industrial wastewater represents a key strategy to mitigate global challenges such as resource scarcity and excessive wastewater generation, particularly in the pharmaceutical and chemical industries, aligning with the goals of sustainable development [1, 2]. Consequently, achieving efficient solute–solute selective separation offers a promising strategy for industrial water treatment, enabling simultaneous wastewater reuse and recovery of valuable resources [2, 3, 4]. Nanofiltration (NF) membranes offer a green, low‐energy, and easily scalable platform with minimal chemical consumption for the selective separation of charged solutes in aqueous systems [5, 6, 7]. For instance, NF membranes are applied for sulfate removal in desalination pretreatment and selective ion fractionation (Mg^2+^, Ca^2+^) in industrial brines [8, 9]. In addition, NF membranes have received growing attention in sustainable direct lithium extraction technologies, where the selective separation of lithium ions (Li^+^) from high‐salinity brines remains a critical challenge [10, 11, 12].

Typically, NF membranes are fabricated as thin‐film composite (TFC) structures, in which an ultrathin polyamide (PA) selective layer is formed on a porous support via interfacial polymerization (IP) reaction between aqueous amines and organic acyl chlorides [13, 14]. The characteristics of the PA layer are critical for charged‐solute separations, especially for mono/divalent ion discrimination. In general, ion transport through NF membranes is primarily governed by a combination of steric sieving and electrostatic interactions [15, 16]. However, most laboratory‐fabricated and commercial NF membranes exhibit a single dominant surface charge, either negative or positive, causing electrostatic interactions to dominate ion selectivity [17, 18, 19, 20, 21, 22, 23, 24, 25]. Negatively charged membranes typically show strong electrostatic repulsion toward multivalent anions (e.g., SO_4_^2−^), resulting in high rejection of SO_4_^2−^ while allowing relatively facile transport of monovalent anions such as Cl^−^, enabling effective mono/divalent anion separations (e.g., Cl^−^/SO_4_^2−^) [24, 26]. Membranes preferentially repel multivalent cations (e.g., Mg^2+^), resulting in higher rejection of divalent cations than monovalent ones and enabling separations such as Li^+^/Mg^2+^ fractionation [27, 28, 29, 30]. Overall, these single‐charged NF membranes tend to exhibit selectivity toward co‐ions with the same charge sign, whereas the separation of counter‐ions is often limited, making their separation performance highly dependent on specific ion systems.

This limitation becomes even more pronounced in practical water treatment, where industrial wastewaters commonly contain complex mixtures of coexisting ionic species [31, 32]. Competitive transport, charge screening, and ion−ion interactions under such conditions significantly reduce the effectiveness of charge‐dominated membranes, making it difficult to simultaneously achieve high rejection of divalent ions and efficient permeation of monovalent species [25]. To address this challenge, zwitterionic modification has emerged as an effective strategy to regulate membrane surface charge. Zwitterionic groups contain both positive and negative charges within the same molecular unit, enabling simultaneous interactions with cations and anions [33, 34, 35, 36, 37, 38] and thereby weakening charge‐sign‐dependent electrostatic effects. When incorporated into NF membranes, such interfaces can suppress excessive Donnan exclusion and shift ion transport toward a more sterically dominated separation regime.

Compared with small‐molecule modifiers, zwitterionic copolymers provide multiple anchoring sites for stable grafting and greater structural tunability, as their reactive segments determine grafting density while zwitterionic segments contribute to surface charge neutralization. Previous study has demonstrated that grafting zwitterionic copolymers onto PA membranes via secondary interfacial reactions can generate near‐neutrally charged surfaces and improve mono/divalent anion selectivity (Cl^−^/SO_4_^2−^) [39]. However, the internal balance between the reactive anchoring segments, which enable covalent attachment and structural reconstruction of the PA layer, and zwitterionic segments, which provide surface charge neutralization, has not been systematically explored. Because the former governs grafting density and potential pore structure evolution while the latter determines charge compensation, regulating their relative composition offers a promising route to precisely tune membrane surface charge and pore structure, thereby optimizing the interplay between electrostatic exclusion and steric sieving.

**FIGURE 1:**
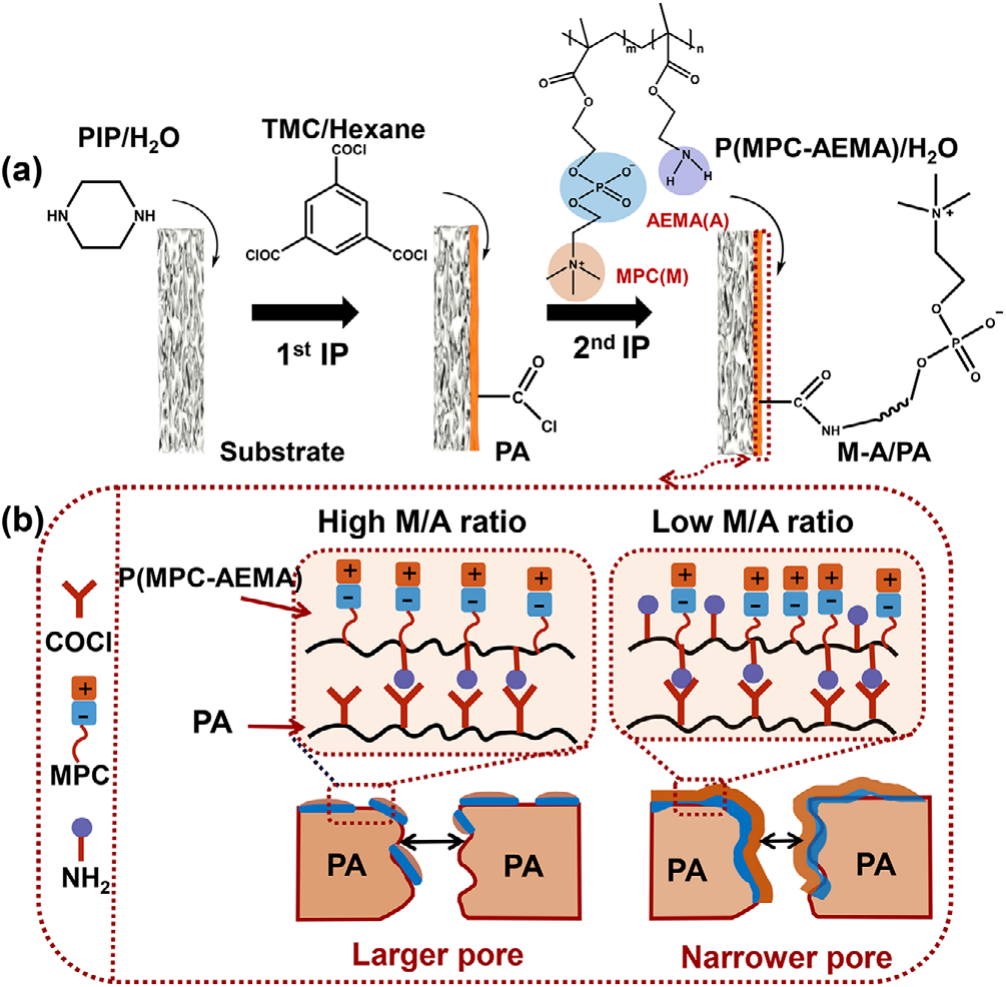
(a) M‐A/PA membrane preparation process and the chemical structure of P(MPC–AEMA) copolymer. (b) Schematic diagram of the changes in membrane grafting as the high or low M/A ratio in P(MPC–AEMA) copolymer.

Herein, we propose a composition‐regulated zwitterionic copolymer grafting strategy to precisely control membrane surface charge and pore structure (Figure 1a,b). By systematically tuning the molar ratio between 2‐methacryloyloxyethyl phosphorylcholine (MPC) and 2‐aminoethyl methacrylate hydrochloride (AEMA) in the copolymer poly(2‐methacryloyloxyethyl phosphorylcholine‐co‐2‐aminoethyl methacrylate hydrochloride) (P(MPC–AEMA)), the grafting density during the secondary IP process can be effectively regulated, thereby modulating both the surface charge distribution and the pore size of the PA selective layer. The reactive AEMA segments provide amine groups that promote covalent grafting and structural reconstruction of the PA selective layer, whereas the zwitterionic MPC segments contribute to charge neutralization. Consequently, the actual density of zwitterionic functionalities introduced onto the membrane surface, as well as the resulting pore structure, depends on the balance between these two segments. At a high MPC/AEMA ratio, the limited number of reactive amine groups leads to a relatively low grafting density, resulting in a less continuous grafted layer, larger effective pore size, and a nearly neutral membrane surface. In contrast, a low MPC/AEMA ratio promotes more extensive grafting, leading to a more continuous selective layer with reduced pore size and a slightly increase in surface charge (Figure 1b). Systematically regulating their ratio is therefore essential for understanding and controlling the coupled effects of surface charge and steric structure in NF membranes.

## 2 Results and Discussion

### 2.1 Chemical Structure Characterization of P(MPC–AEMA) Copolymers

Prior to membrane fabrication, zwitterionic copolymers with different MPC/AEMA (M/A) feed molar ratios (5/5, 7/3, and 9/1) were selected and systematically characterized by Fourier‐transform infrared spectroscopy (FTIR) and ^1^H nuclear magnetic resonance (^1^H NMR). FTIR spectra (Figure S1) were used to qualitatively verify the compositional variation of the copolymers. The characteristic bands at 1240, 1090, and 970 cm^−1^ associated with the phosphorylcholine group of the MPC segment progressively decreased as the M/A feed molar ratio decreased from 9/1 to 5/5, whereas the intensity related to the amine groups in the AEMA segment increased correspondingly [40, 41]. These trends qualitatively indicate the changes of MPC and AEMA contents in the copolymer.

**FIGURE 2:**
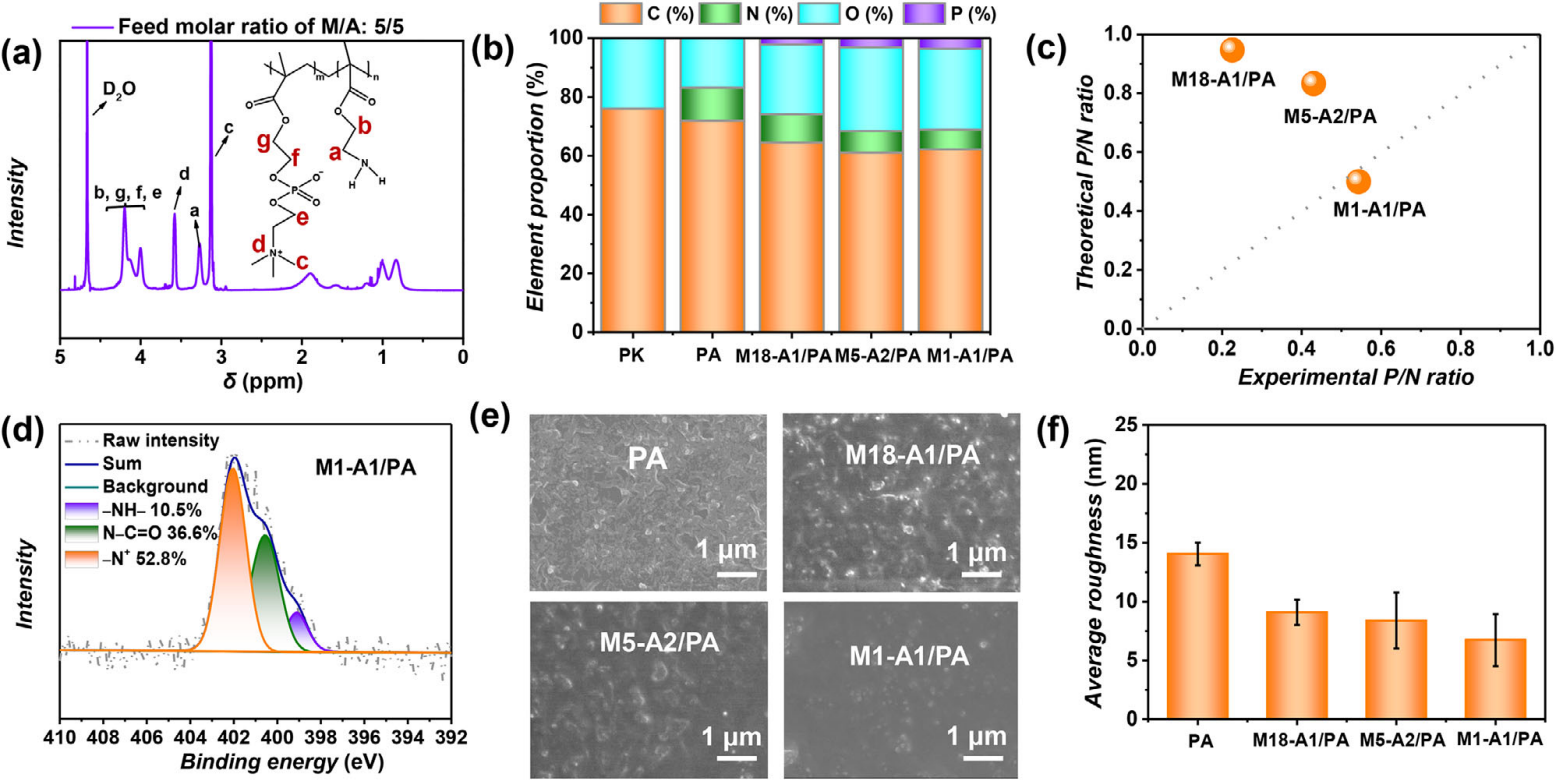
(a) Enlarged ^1^H nuclear magnetic resonance (NMR) spectra of the P(MPC–AEMA) copolymer with a feed molar ratio of M/A (5/5). (b) Element composition of the PK substrate, PA, M18‐A1/PA, M5‐A2/PA, and M1‐A1/PA membranes, respectively, and (c) theoretical and experimental P/N ratio of M18‐A1/PA, M5‐A2/PA, and M1‐A1/PA membranes. (d) High‐resolution N1s spectra of the M1‐A1/PA membrane; (e) SEM images and (f) average roughness of the PA, M18‐A1/PA, M5‐A2/PA, and M1‐A1/PA membranes, respectively. The data in f are shown as mean ± SD, n = 3. The preparation conditions for the Mx‐Ay/PA membrane involve using a PIP solution at 0.5 wt.% and a trimesoyl chloride TMC solution at 0.1 wt.% for the fabrication of PA, along with an Mx‐Ay modification solution at 1.0 wt.%.

The copolymer composition was further analyzed by ^1^H NMR spectroscopy (Figure 2a and Figure S2a,b). The resonance peak labeled a, corresponding to the methylene protons adjacent to the amine group in the AEMA segment, and peak c, assigned to the ─N^+^(CH_3_)_3_ groups of the MPC segment, were consistent with previously reported assignments [42, 43]. By integrating these characteristic peaks, the actual M/A ratios were determined to be approximately 18/1, 5/2, and 1/1, for feed molar ratios of 9/1, 7/3, and 5/5, respectively. These results agree well with the qualitative trends observed in the FTIR analysis. Overall, adjusting the M/A feed ratio effectively modulated the chemical composition of the resulting zwitterionic copolymers, providing a controllable basis for subsequent regulation of grafting behavior and membrane surface properties.

### 2.2 Fabrication and Chemical Characterization of PA Membranes

The pristine PA membranes were first fabricated via conventional IP process between piperazine (PIP) and trimesoyl chloride (TMC) on a porous polyketone (PK) substrate. Zwitterionic copolymers with different M/A ratios were subsequently introduced via an in situ secondary IP process. During this process, the primary amine groups in the AEMA segments reacted with residual acyl chloride groups on the nascent PA surface, enabling covalent grafting of the copolymer. The resulting membranes were denoted as Mx‐Ay/PA (M18‐A1/PA; M5‐A2/PA; M1‐A1/PA), where x and y represent the actual molar ratios of MPC and AEMA segments determined from the ^1^H NMR analysis.

The evolution of surface chemical composition was systematically analyzed by FTIR and X‐ray photoelectron spectroscopy (XPS). The concentration of grafted copolymer was fixed at 1.0 wt.%, as no significant change in surface composition was observed beyond 1.0 wt.% (Figure S3). According to FTIR spectra, the characteristic peaks of MPC, as assigned in the previous section, appeared after the secondary IP process (Figure S4), confirming successful grafting of the zwitterionic copolymer onto the PA surface [41].

XPS analysis was further used to quantify the surface compositional evolution with varying M/A ratios (Figure S5 and Figure 2b). Because phosphorus (P) originates exclusively from the MPC segments whereas nitrogen (N) exists in both the copolymer and the underlying PA layer, the surface P/N ratio can be used as an indicator of the relative coverage of the grafted copolymer. As the M/A ratio decreased from 18/1 to 1/1, the surface phosphorus content increased while the nitrogen content gradually decreased, resulting in a progressively higher P/N ratio.

To evaluate the grafting coverage, the experimentally measured P/N ratios of the membrane surface were compared with the theoretical values calculated from the copolymer compositions (Figure 2c). If the membrane surface was completely covered by the grafted copolymer, the experimental P/N ratio would approach the theoretical value. Accordingly, the M18‐A1/PA and M5‐A2/PA membranes exhibited significantly lower P/N ratios than their corresponding theoretical values, indicating incomplete copolymer coverage. In contrast, the M1‐A1/PA membrane showed good agreement between the experimental and theoretical P/N ratios, suggesting nearly complete surface coverage by the grafted copolymer. This discrepancy mainly originates from the contribution of nitrogen species from the underlaying PA layer. At higher M/A ratios, the limited availability of reactive amine groups restricts the grafting reaction, leaving portions of the PA surface exposed. Consequently, the abundant amine groups in the PA layer dominate the surface N signal detected by XPS, leading to a lower P/N ratio. In contrast, decreasing the M/A ratio increases the availability of amine groups in the copolymer, promoting more extensive grafting during the secondary IP process and yielding a P/N ratio closer to the theoretical value.

High‐resolution N1s spectra further support this interpretation (Figure 2d and Figure S6). Compared with the pristine PA membrane, a new peak assigned to quaternary ammonium (─N^+^) species appeared at approximately 402 eV in the Mx‐Ay/PA membranes, confirming the presence of MPC [44, 45]. The M18‐A1/PA membrane exhibited the lowest fraction of ─N^+^ species and the highest fraction of free ─NH groups, indicating limited grafting and a membrane surface dominated by the underlying PA layer. In contrast, M1‐A1/PA membrane presented an increased fraction of both ─N^+^ and ─NH groups, suggesting higher MPC incorporation with additional amine groups originating from AEMA. These results collectively demonstrate that tuning the M/A ratio effectively regulates the grafting coverage and surface chemical composition of the PA membranes.

### 2.3 The Surface Morphology of PA Membranes

Since the grafting coverage of the zwitterionic copolymer varies with the M/A ratio, the structural reconstruction of the PA layer is expected to influence the resulting membrane surface morphology. To investigate this effect, field emission scanning electron microscopy (FE‐SEM) and atomic force microscopy (AFM) were employed. Compared with the pristine PA membrane (Figure 2e), the M18‐A1/PA membrane exhibited a more compact but discontinuous multi‐granular surface, possibly due to the limited degree of secondary IP reaction. As M/A ratio decreased (M5‐A2/PA), more pronounced nodular structures gradually appeared, suggesting enhanced interfacial reactions between additional amine groups and residual acyl chloride groups on the PA surface. When the M/A ratio further decreased to 1/1 (M1‐A1/PA), the membrane surface became smoother and more continuous, which was attributed to the higher grafting density and thus more complete coverage of the PA surface by the grafted copolymer. This morphological evolution is consistent with the XPS results. AFM results further confirmed this trend, with surface roughness decreasing sequentially from the pristine PA membrane to M18‐A1/PA and M5‐A2/PA and reaching the lowest value for M1‐A1/PA (Ra = 14.04 ± 0.96, 9.08 ± 1.08, 8.39 ± 2.38, and 6.73 ± 2.21 nm, respectively) (Figure S7 and Figure 2f).

Water contact angle (WCA) measurements were further performed to evaluate the surface wettability (Figure S8). Compared with the pristine PA membrane, the modified membranes did not show a significant decrease in WCA after zwitterionic grafting. This is because the pristine PA membrane already contains hydrophilic carboxyl and amine groups. Moreover, the grafted zwitterionic side chains might not be fully exposed at the membrane surface but partially distributed within the selective layer, resulting in only a slight change in WCA.

### 2.4 Effect of Physicochemical Properties of Membranes on Separation Performance

**FIGURE 3:**
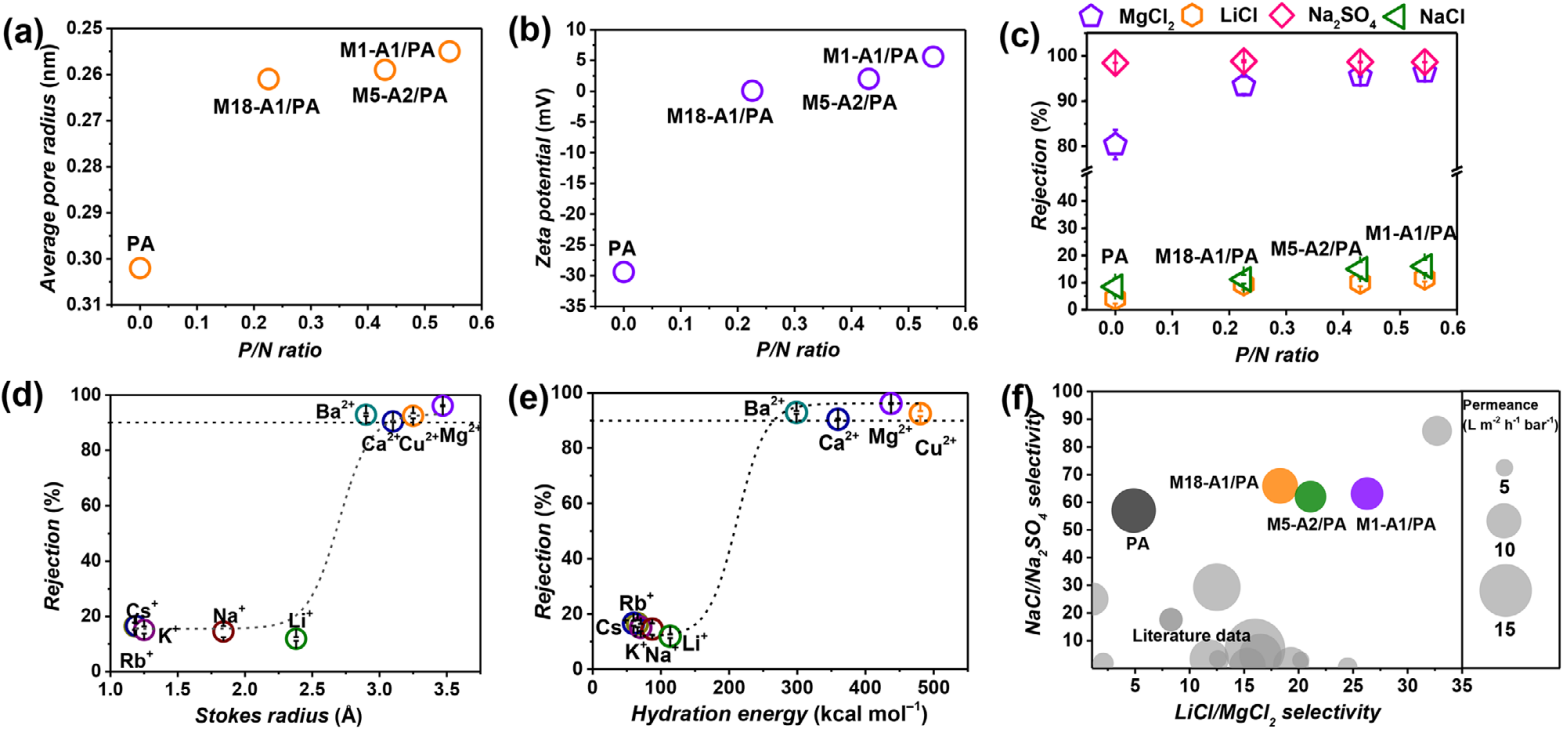
The correlation of P/N ratio of PA membrane with (a) average pore size and (b) zeta potential. (c) MgCl_2_, LiCl, Na_2_SO_4_ and NaCl rejection (Feed solution: each 2000 ppm single salt solution). The rejection of cations of M1‐A1/PA membrane as (d) a function of stokes radius and (e) hydration energy (The details are presented in Table S1). (f) Comparison of NaCl/Na_2_SO_4_ selectivity, LiCl/MgCl_2_ selectivity, and water permeance between the membranes reported in the literature and membranes prepared in this work (the details are presented in Table S2). The data in c, d, and e are shown as mean ± SD, n = 3.

To understand the relationship between membrane physicochemical properties and separation performance, the P/N ratio obtained from XPS was used as an indicator of copolymer grafting density. Specifically, both average pore size (derived from molecular weight cut‐off measurements, Figure S9) and surface zeta potential (Figure S10) exhibit strong correlations with the P/N ratio (Figure 3a,b). The pristine PA membrane exhibits the largest average pore radius (∼0.30 nm) and a strongly negative surface potential (approximately −30 mV). As the P/N ratio increases, the pore radius gradually decreases (from 0.3 nm to 0.26 nm) due to increasing coverage of the zwitterionic copolymer. Simultaneously, the membrane strongly negative toward near‐neutral values (from −30 to ∼0 mV) as the oppositely charged groups within the zwitterionic copolymer neutralize surface charge. In the M1‐A1/PA membrane, which exhibited complete surface coverage, the surface potential was nearly neutral (∼5 mV). The slight positive shift can be attributed to residual unreacted amine groups from the AEMA segments.

The properties of modified membranes play a pivotal role in determining their separation performance [2, 46]. The single‐salt separation performance of the prepared membranes was evaluated using a cross‐flow system at 5 bar with 2000 ppm MgCl_2_, LiCl, NaCl, and Na_2_SO_4_ feed solutions. As a control experiment, a membrane with a selective layer formed solely from P(MPC–AEMA) was prepared via IP between P(MPC–AEMA) and TMC. This copolymer‐only membrane exhibited a continuous decline in water permeance over time and negligible rejection for all tested salts (Figure S11). Such behavior likely arises from the formation of a loosely crosslinked and highly hydrated polymer network that undergoes compaction under pressure, while lacking sufficiently tight free‐volume structures for effective steric or electrostatic exclusion. Therefore, the grafted P(MPC–AEMA) on the PA surface mainly functions as a surface modification layer that regulates the PA surface properties rather than acting as an additional selective layer.

The Mx‐Ay/PA membranes were fabricated via a secondary IP process following the initial IP between PIP and TMC. The initial IP conditions were first optimized to balance water permeance and selectivity of the pristine PA membrane (Figure S12), and the optimal copolymer grafting concentration was determined to be 1.0 wt.% based on the overall separation performance (Figures S13 and S14). The effect of the M/A ratio on membrane separation performance was then investigated (Figure 3c). As the M/A ratio decreased, MgCl_2_ rejection increased significantly, while Na_2_SO_4_ rejection remained consistently high. Meanwhile, the rejection of NaCl and LiCl remained low but showed a slight increase. This trend is mainly attributed to enhanced steric hindrance resulting from increased grafting, which reduces the effective pore size. In addition, the nearly neutral membrane surface further weakens electrostatic attraction or repulsion toward ions. Consequently, ions with larger hydrated radii (SO_4_^2−^ and Mg^2+^) are preferentially rejected, whereas smaller monovalent ions (Na^+^ and Li^+^) can more readily permeate. The optimal performance was achieved at the M/A ratio of 1:1 (M1‐A1/PA), where the zwitterionic copolymer forms a uniform and complete surface coverage, generating a well‐defined and defect‐minimized selective layer that enables the highest MgCl_2_ rejection without sacrificing Na_2_SO_4_ rejection, while still maintaining low rejection of monovalent salts. These results highlight that zwitterionic copolymer grafting, combined with precise control of copolymer composition, enables fine‐tuning of membrane separation performance.

The M1‐A1/PA membrane also exhibited high rejection (>90%) toward other divalent cation salts, including Ca^2+^, Cu^2+^, and Ba^2+^ (Figure 3d,e and Table S1). In contrast, the rejection of monovalent salts remained low. This behavior can be explained by the relatively small Stokes radius and lower hydration energy of monovalent cations, which allow easier dehydration and transport through the membrane pores [47, 48]. The observed rejection sequence follows the trends of both ion Stokes radius and dehydration energy, indicating that steric sieving dominates the ion separation mechanism in the M1‐A1/PA membrane.

The ideal selectivities of NaCl/Na_2_SO_4_ and LiCl/MgCl_2_ were calculated from their rejection ratios and compared with those of previously reported membranes (Figure 3f and Table S2). The pristine PA membrane showed high NaCl/Na_2_SO_4_ selectivity of 57.0 but poor LiCl/MgCl_2_ selectivity of 4.87, which is typical for negatively charged NF membranes. Most reported membranes similarly show high selectivity for either NaCl/Na_2_SO_4_ or LiCl/MgCl_2_, but rarely for both simultaneously due to their single surface charge characteristics. In contrast, the M1‐A1/PA membrane achieves significantly higher LiCl/MgCl_2_ selectivity (26.3), while maintaining high NaCl/Na_2_SO_4_ selectivity (63.1) with only a slight decrease in water permeance compared with the pristine PA membrane. These results demonstrate that precise regulation of the M/A ratio enables the simultaneous tuning of membrane surface charge and pore size, leading to efficient mono/divalent salt separation.

### 2.5 Separation Performance of Binary, Quaternary, and Realistic Brine Systems

**FIGURE 4:**
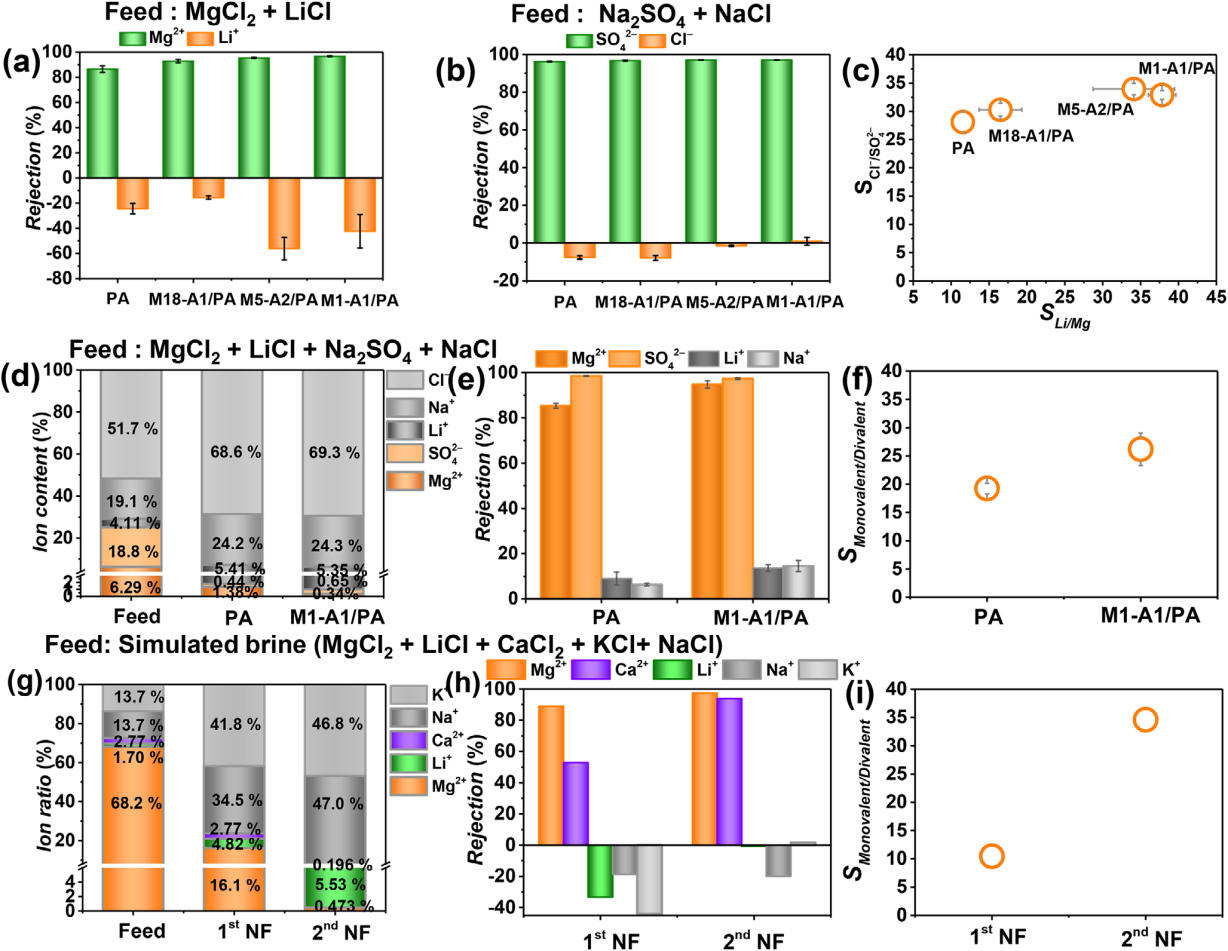
(a) Mg^2+^, Li^+^ rejections (Feed solution: 2000 ppm MgCl_2_ and LiCl mixture solution (Mg^2+^/Li^+^ mass ratio: 20)). (b) SO_4_^2−^, Cl^−^ rejections (Feed solution: mixed solution consisting of NaCl (1g/L) and Na_2_SO_4_ (1g/L)) (c) Cl^−^/SO_4_^2−^ and Li^+^/Mg^2+^ selectivity of PA, M18‐A1/PA, M5‐A2/PA, and M1‐A1/PA membranes, respectively. (d) Ion content in the feed and permeate solutions. (e) Ion rejection and (f) total mono/divalent cation and anion separation selectivity of PA and M1‐A1/PA membranes. (Feed solution: mixed solution consisting of NaCl (1g/L), Na_2_SO_4_ (1g/L), MgCl_2_ (1g/L) and LiCl (1g/L)) (g) ion mass ratio, (h) ion rejection and (i) total mono/divalent separation cation selectivity in the permeate solutions among first and second NF process. (Feed solution: Simulated brine) The data in a, b, c, e and f are shown as mean ± SD, n = 3.

Binary salt mixture solutions were first used to evaluate the mono/divalent ion separation capability of grafted PA membranes. Mixed‐salt experiments were conducted using MgCl_2_/LiCl solutions (2000 ppm, Mg^2+^/Li^+^ mass ratio is fixed at 20) and NaCl/Na_2_SO_4_ solutions (2000 ppm, equal NaCl/Na_2_SO_4_ mass ratio) at 5 bar. Compared with the pristine PA membrane, all Mx‐Ay/PA membranes exhibited markedly enhanced Mg^2+^ rejection while simultaneously maintaining high SO_4_^2−^ rejection. Moreover, Mg^2+^ rejection increased progressively with decreasing M/A ratio, similar with the trends observed in single‐salt experiments (Figure 4a,b). Notably, apparent negative rejection of monovalent ions was observed in the mixed‐salt tests, including Li^+^ in the LiCl/ MgCl_2_ mixture and Cl^−^ in the NaCl/ Na_2_SO_4_ mixture. Taking the LiCl/MgCl_2_ system as an example, Mg^2+^ is strongly rejected by the membrane, whereas Cl^−^ can permeate relatively readily. This preferential passage of Cl^−^ would create a charge imbalance in the permeate. Therefore, additional Li^+^ transport is promoted to maintain electroneutrality. As a result, the Li^+^ concentration in the permeate becomes higher than that in the feed, leading to apparent negative Li^+^ rejection [39, 49]. A similar ion‐coupled transport mechanism also accounts for the negative rejection of Cl^−^ in the NaCl/Na_2_SO_4_ mixture, where strong SO_4_^2−^ rejection promotes additional Cl^−^ transport to balance the permeation of Na^+^.

The mono/divalent separation factors were further calculated to compare the ion selectivity of the pristine PA membrane and the Mx‐Ay/PA membranes (Figure 4c). The pristine PA membrane shows high mono/divalent anion selectivity (Cl^−^/SO_4_^2−^ = 30.0) but relatively low mono/divalent cation selectivity (Li^+^/Mg^2+^ = 12.0), consistent with its negatively charged surface and relatively large pore size. In contrast, the M1‐A1/PA membrane exhibits the highest Li^+^/Mg^2+^ selectivity (39.8), significantly higher than those of the M18‐A1/PA (16.8) and M5‐A2/PA (31.1) membranes, while still maintaining high Cl^−^/SO_4_^2−^ selectivity (33.8). This favorable performance can be attributed to the finely controlled pore size and the near‐neutral surface by complete zwitterionic copolymer surface coverage.

It is well recognized that the high Mg^2+^ concentration and Mg^2+^/Li^+^ mass ratios in natural salt‐lake brines pose a major challenge to efficient lithium recovery [50]. The M1‐A1/PA membrane exhibited stable Li^+^/Mg^2+^ separation performance across a range of Mg^2+^/Li^+^ ratios (Figure S15a,c) and salinities, with only a slight decline at higher concentrations due to increased osmotic pressure (Figure S15b,d) [48, 51]. Furthermore, the M1‐A1/PA membrane showed a good operational stability, maintaining nearly constant water permeance and ion rejection during a seven‐day continuous filtration test (Figure S16). Compared with previously reported membranes, the M1‐A1/PA membrane achieves a favorable balance between high Li^+^ permeance and Li^+^/Mg^2+^ separation selectivity through precise M/A ratio regulation. Such a combination enables efficient lithium transport while maintaining high lithium product purity, underscoring its great potential for lithium extraction [10, 11] (Figure S17 and Table S3).

Performance tests of Mx‐Ay/PA membranes were further extended to quaternary feed salt mixtures. A feed solution containing MgCl_2_, LiCl, Na_2_SO_4_, and NaCl (1 g/L each) was prepared to compare the separation performance of the pristine PA and M1‐A1/PA membranes toward multivalent and monovalent ions. This quaternary salt system was employed as a model mixed‐ion feed to evaluate the influence of ion coexistence on the membrane separation performance, providing a more realistic assessment of its potential for application in complex aqueous systems. The ion compositions of the feed and permeate solutions are summarized in Figure 4d. In the pristine PA membrane, SO_4_^2−^ exhibited strong rejection, with its proportion decreasing from 18.8% in the feed to 0.44% in the permeate due to the electrostatic repulsion from the negatively charged membrane surface. Mg^2+^ also possessed certain rejection, with its proportion decreasing from 6.29% to 1.38%. By comparison, the M1‐A1/PA membrane effectively rejected both Mg^2+^ and SO_4_^2−^ (Figure 4e), resulting in permeate fractions of only 0.34% and 0.65%, respectively, while allowing free passage of monovalent ions (Li^+^ and Cl^−^). This behavior is further supported by the significantly enhanced total mono/divalent cation and anion selectivity compared to the pristine PA membrane (Figure 4f). These results confirm that the M1‐A1/PA membrane enables efficient charge‐sign‐independent mono/divalent ion sieving in complex multi‐ion systems.

To further assess its practical applicability, a more realistic multi‐ion feed solution simulating the Taijinar salt‐lake brine was prepared. A two‐stage separation process was applied to improve the Li^+^ enrichment, where the permeate from the first NF process was directly used as the feed for the second stage. The evolution of ion ratios during the two‐stage NF process is summarized in Figure 4g. After the second NF stage, the Mg^2+^ ratio drops dramatically from 68.2% to 0.473%, while the Li^+^ ratio rises from 2.77% to 5.53%, indicating efficient lithium enrichment. As shown in Figure 4h, the membrane preferentially rejects divalent cations (Mg^2+^ and Ca^2+^), followed by selective permeation of monovalent ions (Li^+^, Na^+^, and K^+^), exhibiting a clear mono/divalent separation behavior. As a result, the overall mono/divalent selectivity is markedly improved after second NF process, as demonstrated in Figure 4i. The simultaneous removal of Mg^2+^ and enrichment of Li^+^ highlights the strong potential of the M1‐A1/PA membrane for lithium extraction from complex salt‐lake brines. Embedding this membrane process into direct lithium extraction systems could offer significant advantages over conventional evaporation pond methods, including faster processing, lower resource consumption, and more efficient lithium recovery from highly complex brine systems [52]. Nevertheless, future efforts may focus on further optimizing zwitterionic grafting strategies and surface charge regulation to improve antifouling properties and facilitate the practical application of ion‐selective NF membranes.

## 3 Conclusions

In this work, we demonstrate that regulating the composition of grafted zwitterionic copolymers provides an effective and rational strategy to simultaneously tailor the surface charge and pore structure of NF membranes. By tuning the molar ratio of MPC to AEMA, the balance between reactive anchoring and charge‐neutralizing segments can be precisely controlled. A lower M/A ratio increases the density of reactive AEMA sites, leading to enhanced grafting, reduced effective pore size, and the formation of a near‐neutral membrane surface. Compared with the negatively charged PA membrane, the Mx‐Ay/PA membranes achieve high rejection of divalent ions (e.g., Mg^2+^ and SO_4_^2−^), while maintaining low rejection of monovalent ions (e.g., Na^+^, Li^+^), indicating a transition from charge‐dominated to sterically governed ion transport. Notably, the M1‐A1/PA membrane exhibits outstanding separation performance, delivering a Li^+^/Mg^2+^ selectivity of 39.8 and a Cl^−^/SO_4_^2−^ selectivity of 33.8, while maintaining superior charge‐sign‐independent mono/divalent ion discrimination in multicomponent salt systems. Furthermore, in a two‐stage NF process, the M1‐A1/PA membrane demonstrates strong potential for lithium enrichment from complex brine systems. Overall, this work establishes zwitterionic copolymer composition regulation as a versatile and predictive membrane design strategy, providing new insights into the rational control of membrane structure and function for high‐performance ion‐selective separations in complex aqueous environments.

## 4 Experiment Section

### 4.1 Chemicals and Materials

PK (Molecular weight (Mw): 2 000 000 g/mol) was supplied by Asahi Kasei Corp., Tokyo, Japan. The poly (2‐methacryloyloxyethyl phosphorylcholine‐co‐2‐aminoethyl methacrylate hydrochloride) random copolymers P(MPC–AEMA) with various feed molar ratio of MPC and AEMA (M/A: 9/1, 7/3, and 5/5) were provided by NOF CORPORATION (Tokyo, Japan). TMC was purchased from Sigma–Aldrich (Japan). Anhydrous PIP and hexane were purchased from FUJIFILM Wako Pure Chemical Co., Tokyo, Japan. Deionized water was produced in the lab using a Milli‐Q Integral 3 system (Millipore S.A.S., Molsheim, France).

MgCl_2_·6H_2_O, CaCl_2_, Na_2_SO_4_, NaCl, KCl, and LiCl were obtained from FUJIFILM Wako Pure Chemical Co., Tokyo, Japan. These salts were employed for single‐salt and mixed‐salt filtration experiments. Organic solutes such as sucrose and D‐(+)‐glucose (molecular weight: 180 Da) were purchased from Tokyo Chemical Industry Co., Ltd. (Tokyo, Japan), while glycerol and D‐(+)‐raffinose were supplied by FUJIFILM Wako Pure Chemical Corporation (Tokyo, Japan). These compounds were utilized for determining the MWCO of the prepared membranes.

### 4.2 The PK Substrate and NF Membrane Preparation Method

The preparation method of the PK substrate is consistent with the method we previously reported [53]. The uniform PK casting solution (14 wt.%) was carefully cast onto the pre‐prepared non‐woven fabric surface with a 40 µm casting knife. Then, the membrane was placed in a 35 wt.% methanol aqueous solution and transformed into a membrane. Then transfer to pure acetone and hexane solvents for 20 min, respectively. Finally, place the membrane in a fume hood overnight to dry for later use.

The PA membranes were prepared on PK substrate by IP process. Different concentrations of PA aqueous solutions (0.3, 0.5 1.0 wt.%) were immersed on the PK substrate for 2 min. After that, the excess aqueous solution on the surface was removed with an air knife. Then, the TMC/hexane solution (0.1 wt.%) was poured onto the membrane surface for IP process, which lasted for 1 min. After the reaction, the membrane was placed in a 70°C oven for 3 min. Additionally, the process of preparing pure P(MPC–AEMA)/TMC membranes with different concentrations (Feed molar ratio of M/A: 5/5; Concentration: 0.5, 1.0, 1.5 wt.%) was similar with the PA membranes. Here, the immersing time of TMC/hexane solution (0.1 wt.%) was expanded to 10 min, and the heat treatment time was set as 10 min.

The preparation process of the Mx‐Ay/PA membrane was based on the method used to prepare PA membrane, and the specific preparation steps were shown in Figure 1a. 0.5 wt.% PIP aqueous solution was placed on the surface of the PK substrate for 2 min. The excess PIP aqueous solution was removed and poured onto 0.1 wt.% TMC/hexane organic phase solution for reaction for 1 min. Then, the excess solution was removed, and the Mx‐Ay solution with different concentrations (0.5, 1.0, 1.5 wt.%) and different feed molar ratios of M/A (9/1, 7/3, 5/5) was poured onto the surface of the PA membrane for 10 min for the secondary IP process. After the reaction, the excess aqueous solution was removed, and the membrane was thermally cross‐linked in an oven for 10 min. The prepared Mx‐Ay/PA membrane was stored in deionized water at 4°C for subsequent testing.

### 4.3 Characterization

NMR spectroscopy (ECZ‐400, JEOL, Japan) was used to investigate the chemical structures of the P(MPC–AEMA) copolymers. AFM (SPI3800 N; JEOL, Ltd, Tokyo, Japan) and FE‐SEM (JSF‐7500F, Hitachi Ltd, Tokyo, Japan) were employed to assess the surface structure of the NF membranes. FTIR (ALPHA, Bruker, Billerica, MA, USA) and XPS (JPS‐9010 MC, JEOL, Ltd., Tokyo, Japan) were used to detect the membrane chemical composition. A contact angle goniometer (Drop Master 300; Kyowa Interface Science Co. Ltd., Tokyo, Japan) was used to compare the hydrophilicity of the prepared membranes. The membrane surface charge was evaluated using an electrokinetic analyzer (Anton Paar SurPASSTM 3, Graz, Austria).

### 4.4 NF Membrane Separation Performance Evaluation

The separation performance of the NF membranes was evaluated using a cross‐flow filtration apparatus with an effective membrane area of 7.06 cm^2^ under a fixed pressure of 5 bar. Prior to data collection, the membranes were pre‐pressurized for at least 60 min to achieve permeate mass and conductivity stable, after which water permeance (*P*, L m^−2^ h^−1^ bar^−1^) of the membranes was determined according to the following equation:

(1) $$P=\frac{V}{S\times \Delta t\times p}$$

where *V* (L) is the permeate volume after a filtration time (*∆t*, h), *pta* represents the operation pressure (bar) during the test, and *S* (m^2^) denotes the membrane filtration area. The rejection *R* (%) of the NF membrane was determined using the following equation:

(2) $$R=\left(1-\frac{C_{p}}{C_{f}}\right)\times 100\%$$

where *C_p_* and *C_f_* are concentrations (ppm) in the feed and permeate solutions, respectively. Here, the single salt test concentration is 2000 ppm. The single‐salt ideal selectivity *S_X/Y_* was calculated using Equation (3):

(3) $$S_{X/Y\;}=\left(1-R_{X}\right)/\left(1-R_{Y}\right)$$

where *R_X_* and *R_Y_* are the X and Y slat rejections, respectively. The mixed MgCl_2_/LiCl solution is used to evaluate the Li^+^/Mg^2+^ separation performance of the membrane with different mass ratios (20, 40, 60; Total concentration: 2000 ppm) and different concentrations (2000, 4000, 6000 ppm; Mg^2+^/Li^+^ mass ratios: 20). The mixed Na_2_SO_4_/NaCl solution is used to evaluate the Cl^−^/SO_4_^2−^ separation performance of the membrane with 1g/L of Na_2_SO_4_ and NaCl, respectively. The preparation of the four mixed salt solutions is to use 1g/L of Na_2_SO_4_, NaCl, MgCl_2,_ and LiCl, respectively. The separation factor (*S_M/N_*) was calculated using Equation (4).

(4) $$S_{M\;/\;N}=\frac{C_{p,M}/C_{p,N}}{C_{f,M}/C_{f,N}}\;$$

where *C_f, M_* and *C_p, M_* is the ion M concentrations (ppm) at the feed and permeate solutions, respectively. *C_f, N_* and *C_p, N_* are the ion N concentrations (ppm) at the feed and permeate solution, respectively. The Mg^2+^ and Li^+^ concentrations were measured using an inductively coupled plasma optical emission spectrometer (ICP‐OES; Shimadzu, Japan). The Cl^−^ and SO_4_^2−^ concentrations were tested using the ion chromatograph (IC, ICS‐900, Dionex, USA).

A 10 L simulated Taijinar salt lake solution (183 mg L^−1^ LiCl, 4700 mg L^−1^ MgCl_2_, 609 mg L^−1^ NaCl, 457 mg L^−1^ KCl, and 124 mg L^−1^ CaCl_2_) was formulated as the feed solution for the two‐stage NF process [10, 47]. The system operated under a pressure of 5 bar to obtain a high‐purity lithium stream. The feed solution of the second NF process was the permeate of the first NF process.

### 4.5 MWCO Test

1000 ppm glycerol (92 Da), sucrose (342 Da), (+)‐glucose (180 Da), and D‐(+)‐raffinose (504 Da) aqueous solutions were used to measure the MWCO values and obtain the pore size distribution of the prepared membranes with a constant pressure of 5 bar. The concentrations in feed and permeate solutions were determined by a total organic analyzer (TOC‐VCSH, Shimadzu, Japan). The MWCO values of the membrane were defined as the molecular weight corresponding to 90% solute rejection. The membrane pore size distribution was further evaluated using Equation (5) [54]:

(5) $$\frac{dR\left(d_{p}\right)}{dd_{p}}=\frac{1}{\sqrt{2\pi }d_{p}\ln\sigma_{p}}\;\exp\left[-\frac{{\left(\ln d_{p}-\ln\mu_{p}\right)}^{2}}{2{\left(\ln\sigma_{p}\right)}^{2}}\right]$$

where *d_p_* represents the Stokes radius of each neutral molecule. The mean pore diameter (*μ_p_*) was obtained when the rejection of the neutral solute reached 50%. The geometric standard deviation (*σ_p_*) was derived from the ratio of *d_p_* at a neutral molecule rejection of 84.13% to that at 50%.

### 4.6 Statistical Analysis

All experimental data were presented as means ± standard deviation.

## Author Contributions

**Shaofan Duan**: conceptualization, investigation, formal analysis, Writing – original draft, Writing – review and editing. **Shuai Jiang**: investigation. **Mengyang Hu**: investigation. **Keizo Nakagawa**: writing – review and editing. **Kecheng Guan**: writing – review and editing, conceptualization, supervision. **Feidong Yang**: investigation. **Yongxuan Shi**: investigation. **Tomohisa Yoshioka**: writing – review and editing. **Pengfei Zhang**: investigation. **Yuqing Lin**: investigation. **Eiji Kamio**: writing – review and editing, supervision. **Tae Hoon Lee**: writing – review and editing. **Hideto Matsuyama**: supervision, writing – review and editing. **Yuxuan Feng**: investigation.

## Conflicts of Interest

The authors declare no conflicts of interest.

## Supporting information




**Supporting File**: advs77377‐sup‐0001‐SuppMat.pdf

## Data Availability

The data that support the findings of this study are available from the corresponding author upon reasonable request.
