# Supplementary material for: Precise Control of Charge–Structure Coupling in Polyamide Membranes for Mono‐ and Divalent Ion Separation
Source: Adv Sci (Weinh). 2026 Aug 25:e77377. Online ahead of print. doi: 10.1002/advs.77377 (PMC13505581; doi:10.1002/advs.77377)
Supplement: Supplementary file 1 [file ADVS-9999-e77377-s001.pdf]

Supporting Information

Precise Control of Charge–Structure Coupling in Polyamide Membranes for Mono- and Divalent Ion Separation

Shaofan Duan[a,b], Shuai Jiang[a,b], Feidong Yang[a,b], Yuxuan Feng[b], Yongxuan Shi[a], Pengfei Zhang[a], Mengyang Hu[a], Yuqing Lin[a], Tae Hoon Lee[c], Kecheng Guan[a,*], Keizo Nakagawa[a,d], Tomohisa Yoshioka[a,d], Hideto Matsuyama[a,*], Eiji Kamio[a,b,*]

[a] *Research Center for Membrane and Film Technology, Kobe University, 1-1 Rokkodaicho, Nada, Kobe 657-8501, Japan*
[b] *Department of Chemical Science and Engineering, Kobe University, 1-1 Rokkodaicho, Nada, Kobe 657-8501, Japan*
[c] *Department of Future Energy Engineering, Sungkyunkwan University, Suwon 16419, Republic of Korea*
[d] *Graduate School of Science, Technology and Innovation, Kobe University, 1-1 Rokkodaicho, Nada, Kobe 657-8501, Japan*

Corresponding authors:

Kecheng Guan, e-mail: guan@people.kobe-u.ac.jp

Hideto Matsuyama, e-mail: matuyama@kobe-u.ac.jp

Eiji Kamio, e-mail: e-kamio@people.kobe-u.ac.jp

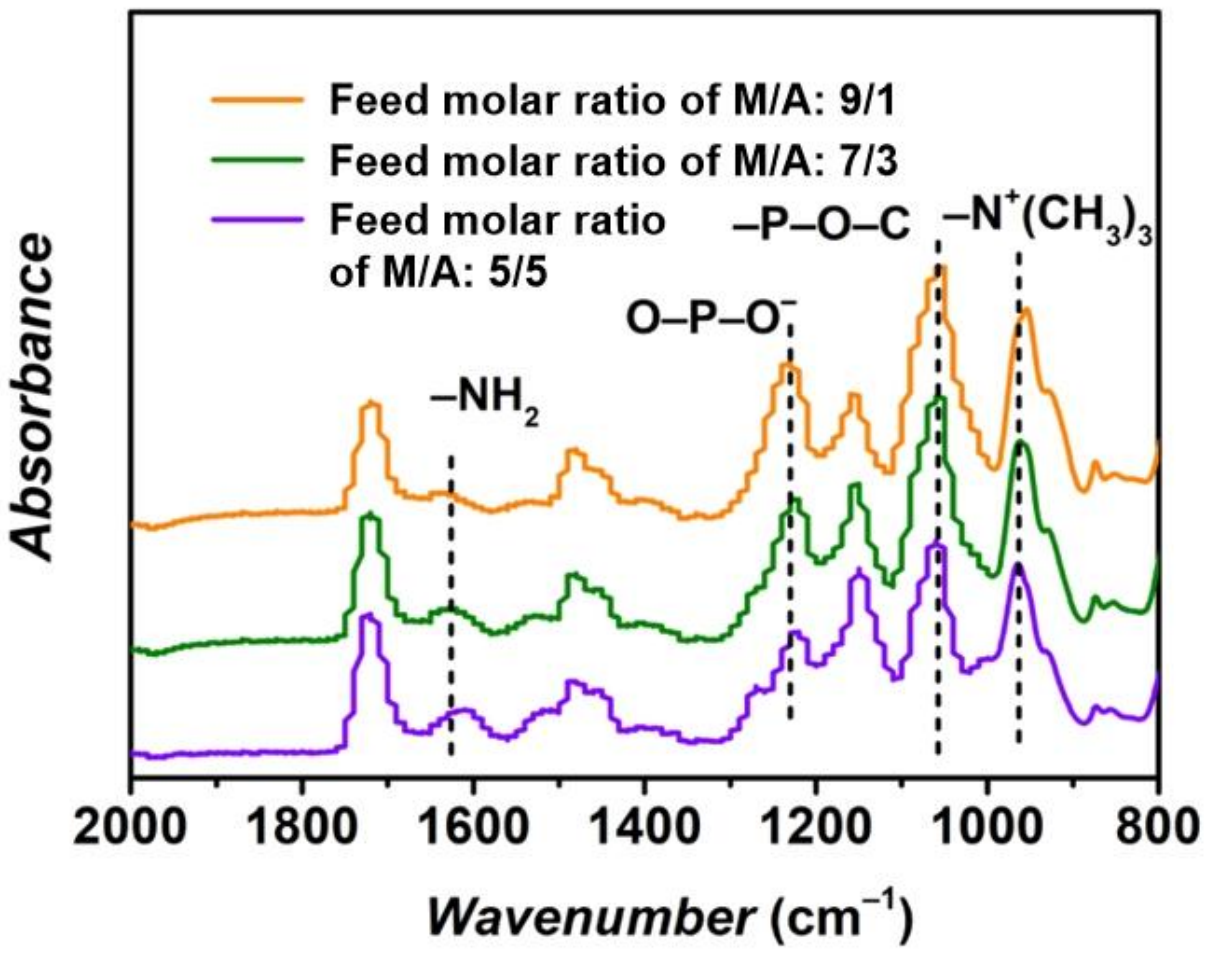

Figure S1. Fourier-transform infrared spectroscopy (FTIR) spectra of P(MPC–AEMA) copolymers with different feed molar ratio of M/A (5/5, 7/3, and 9/1).

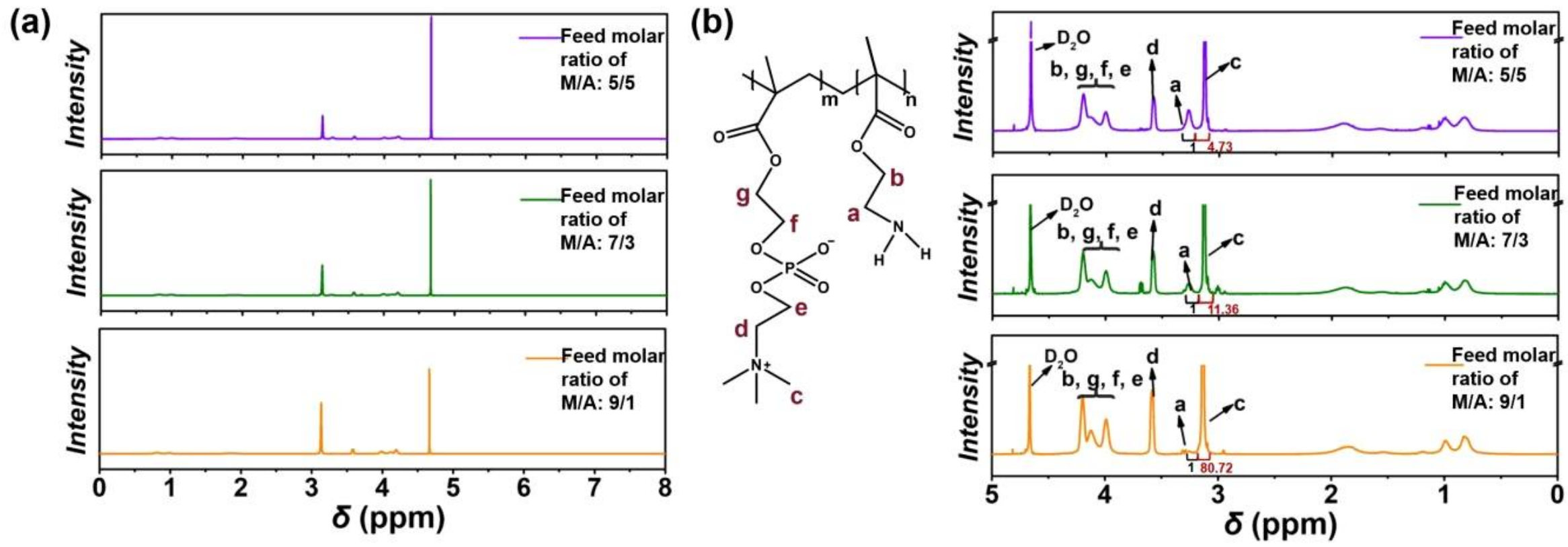

Figure S2. (a) ^{1}H nuclear magnetic resonance (NMR) spectra and (b) Enlarged ^{1}H NMR spectra of P(MPC−AEMA) copolymers with different feed molar ratio of M/A (5/5, 7/3, and 9/1).

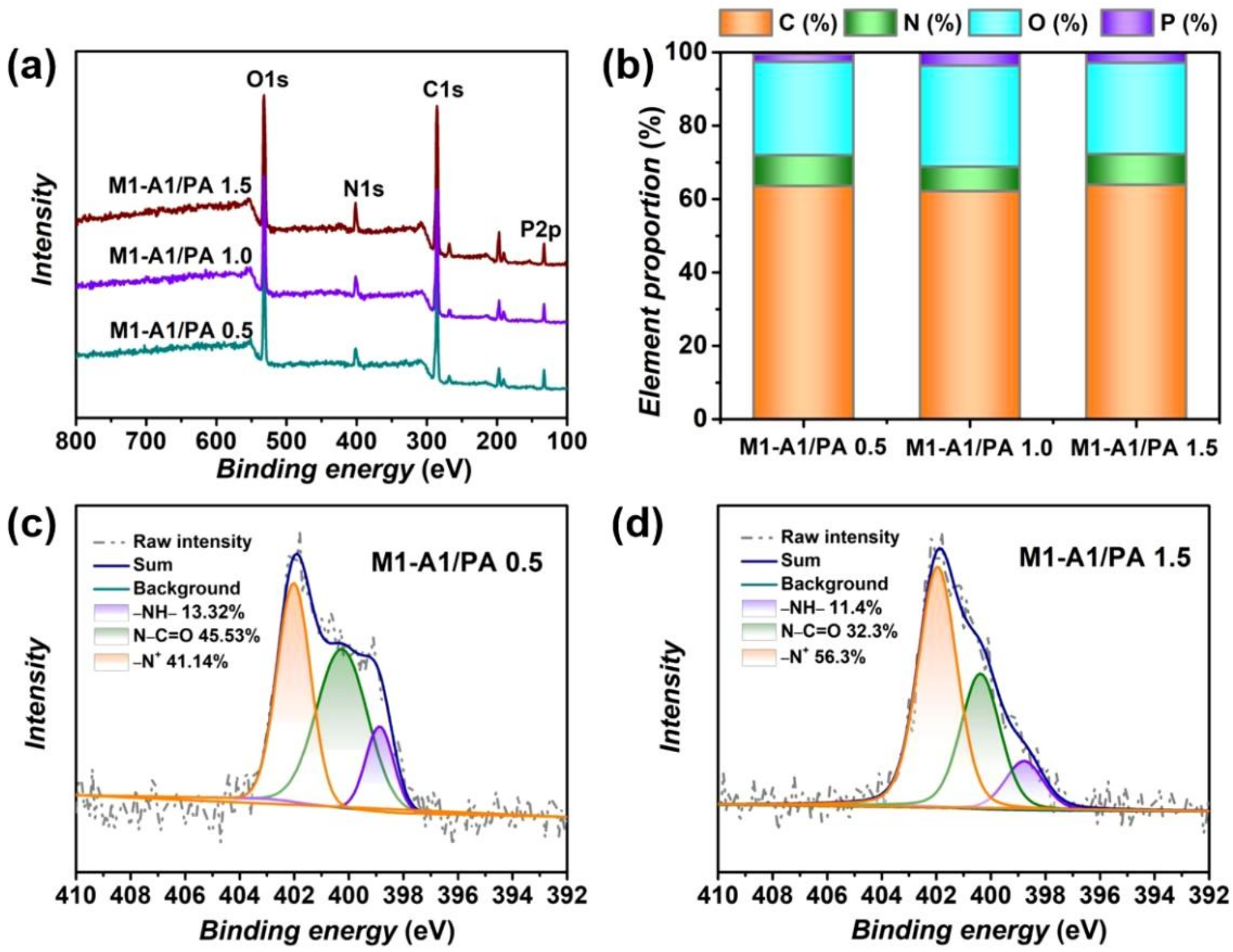

Figure S3. (a) X-ray photoelectron spectroscopy (XPS) spectra and (b) element composition of prepared M1-A1/PA membranes with different concentrations (0.5–1.5 wt%). High resolution N1s spectra of (c) M1-A1/PA 0.5 and (d) M1-A1/PA 1.5 membrane, respectively.

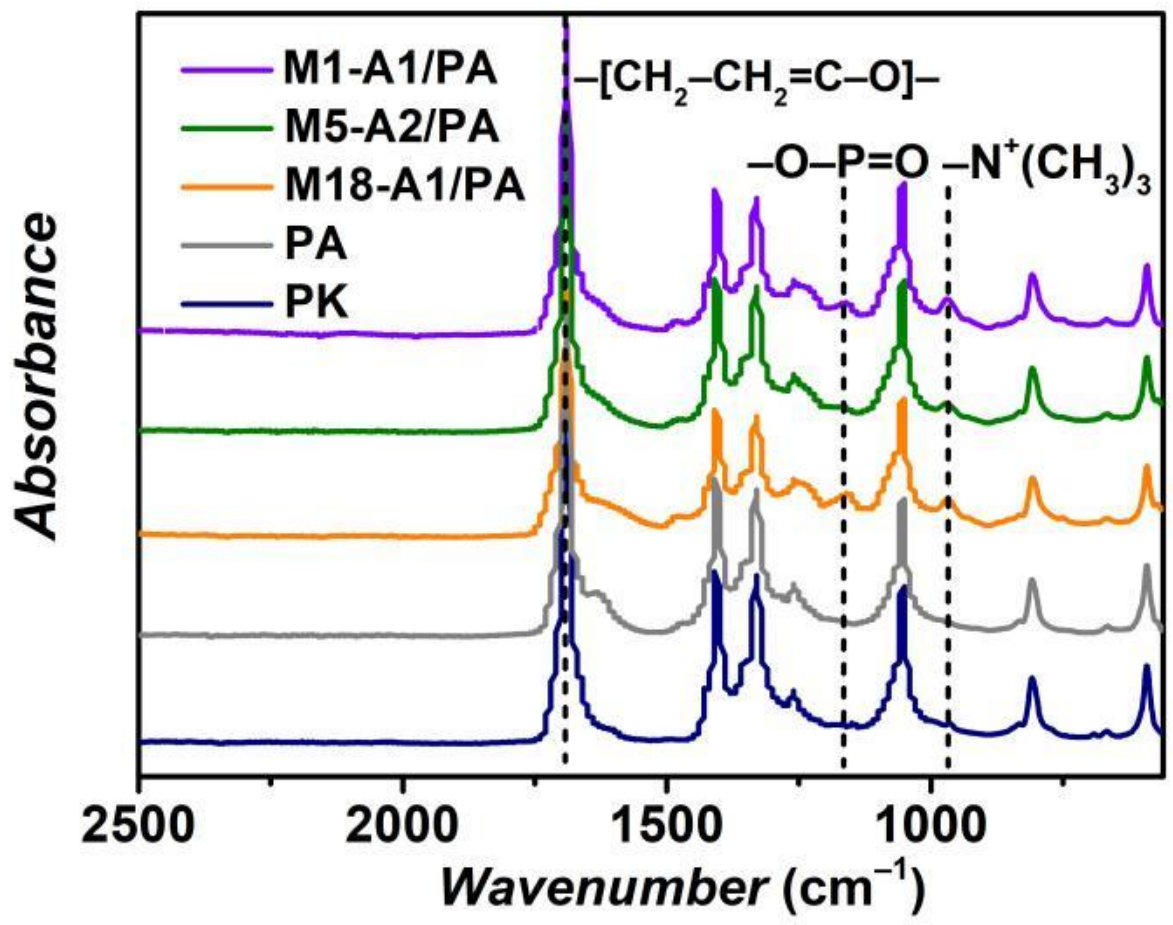

Figure S4. FTIR spectra of PK support and prepared membranes.

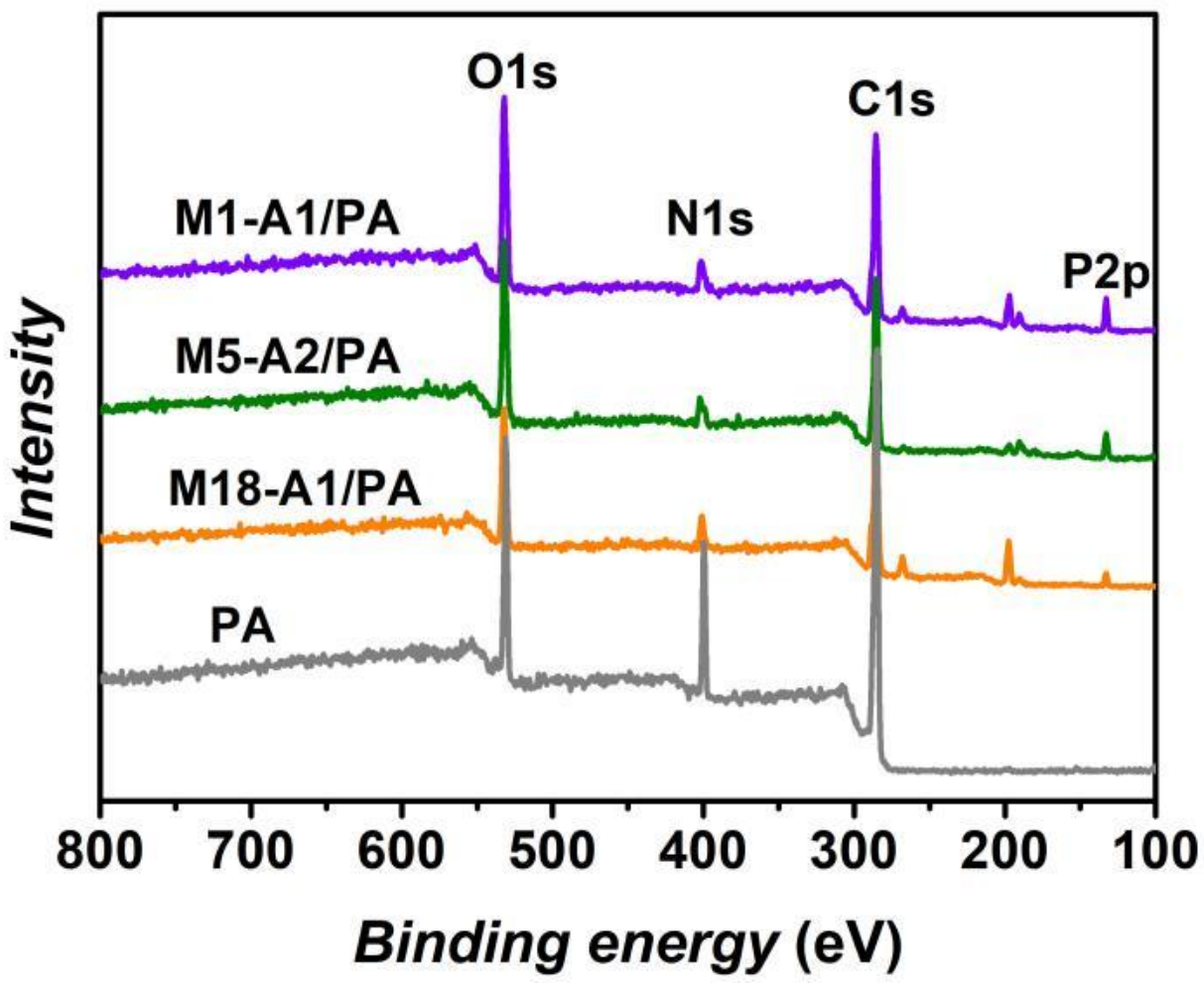

Figure S5. XPS spectra of prepared membranes.

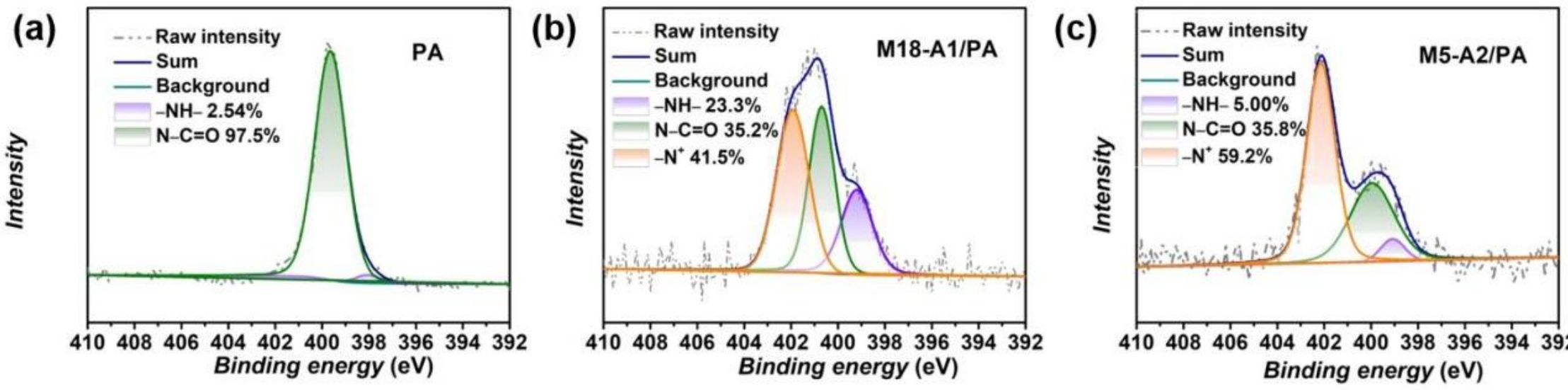

Figure S6. High resolution N1s spectra of (a) PA, (b) M18-A1/PA, (c) M5-A2/PA membranes.

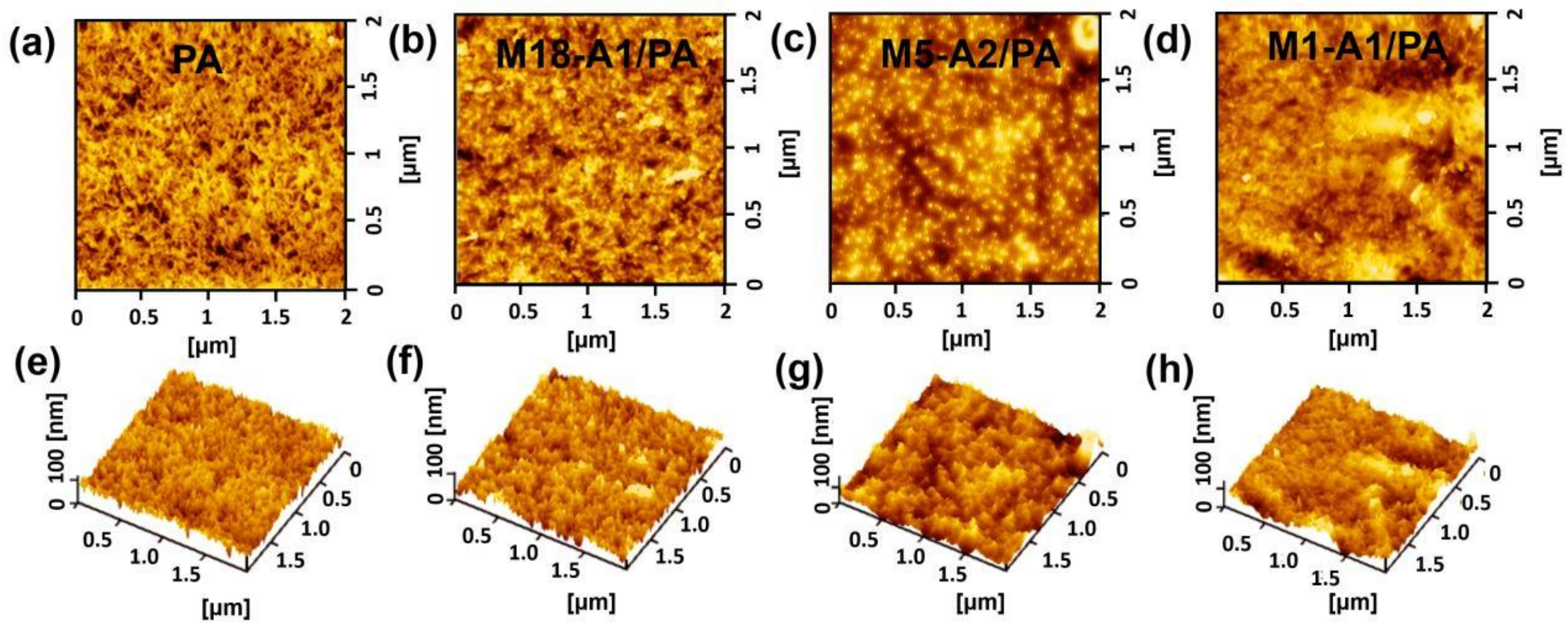

Figure S7. 2D and 3D atomic force microscopy (AFM) images of the (a, e) PA, (b, f) M18-A1/MA, (c, g) M5-A2/MA, and (d, h) M1-A1/MA membranes.

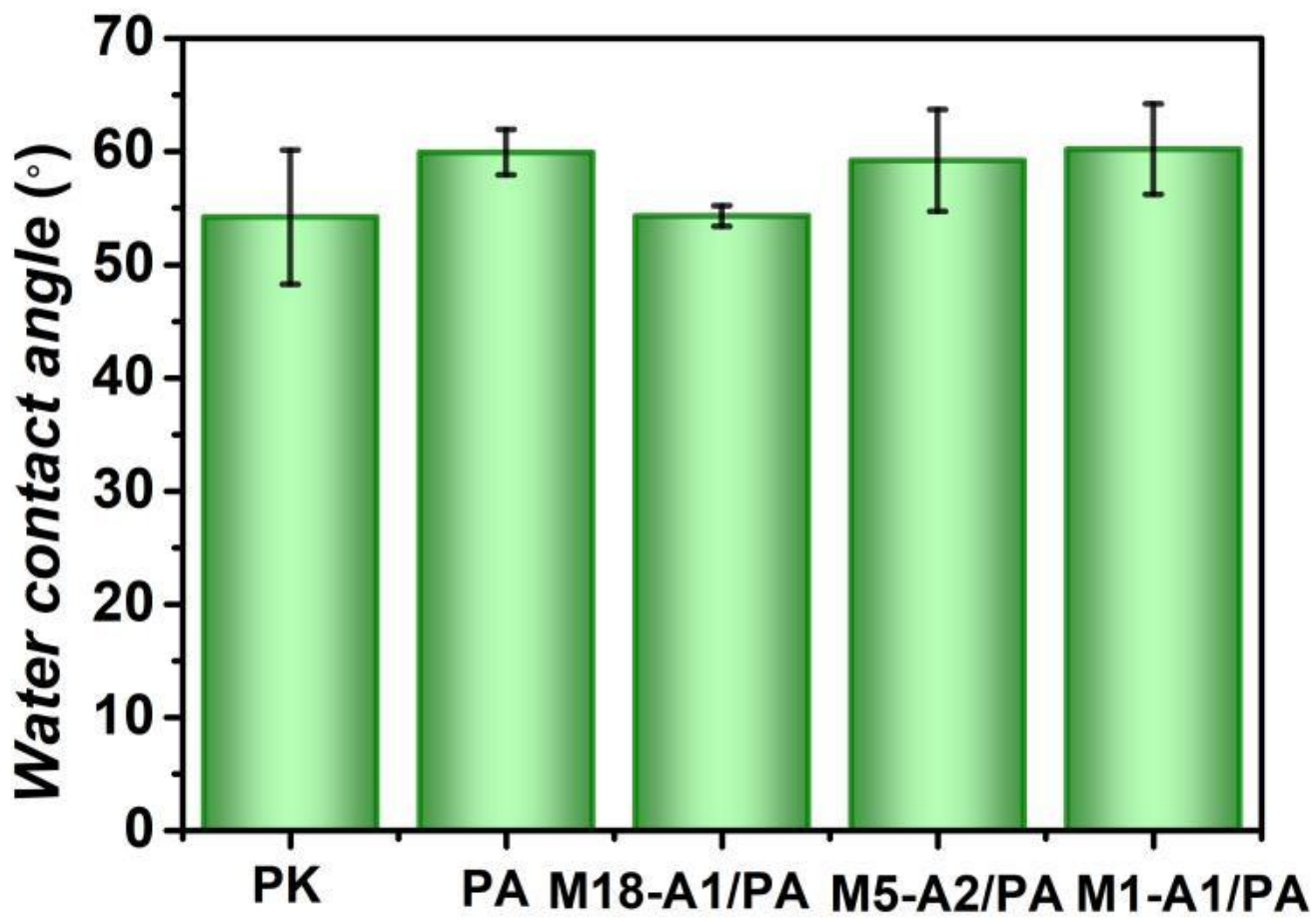

Figure S8. Water contact angle of different membranes. The data are shown as mean ± SD, n=3.

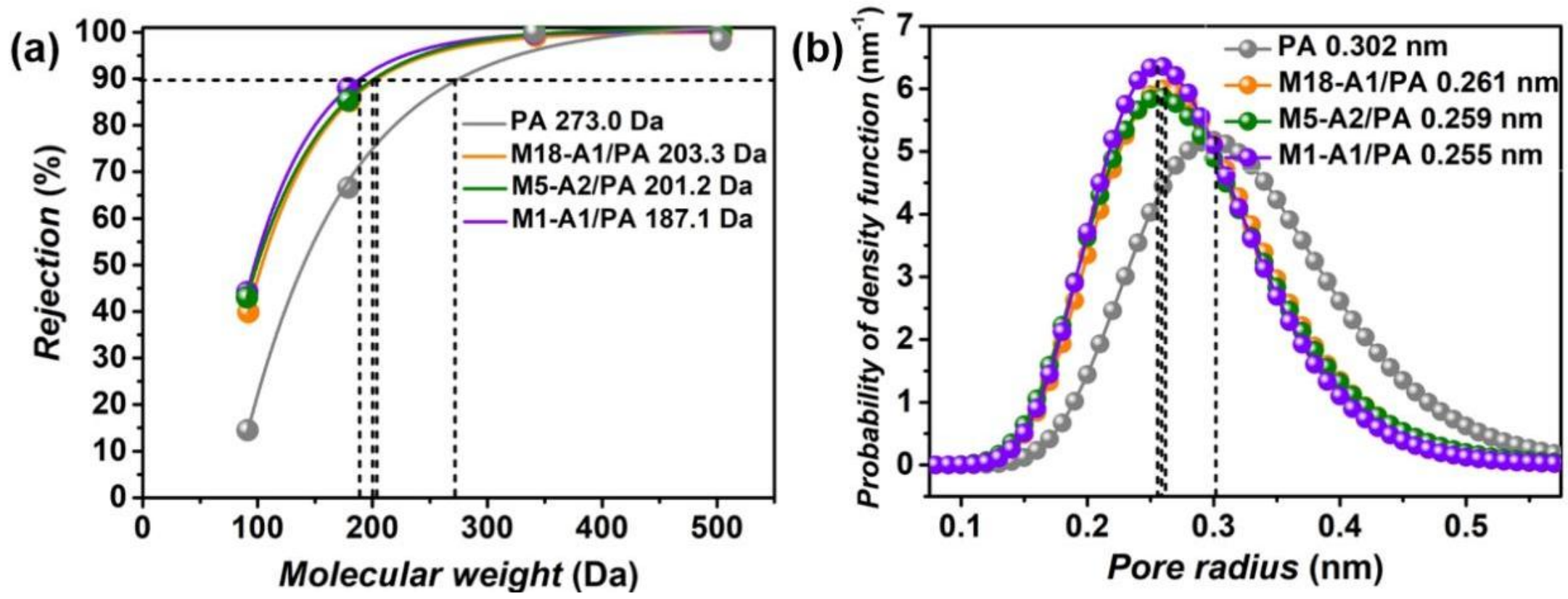

Figure S9. (a) Molecular weight cut-off (MWCO) test and (b) pore size distribution of different membranes.

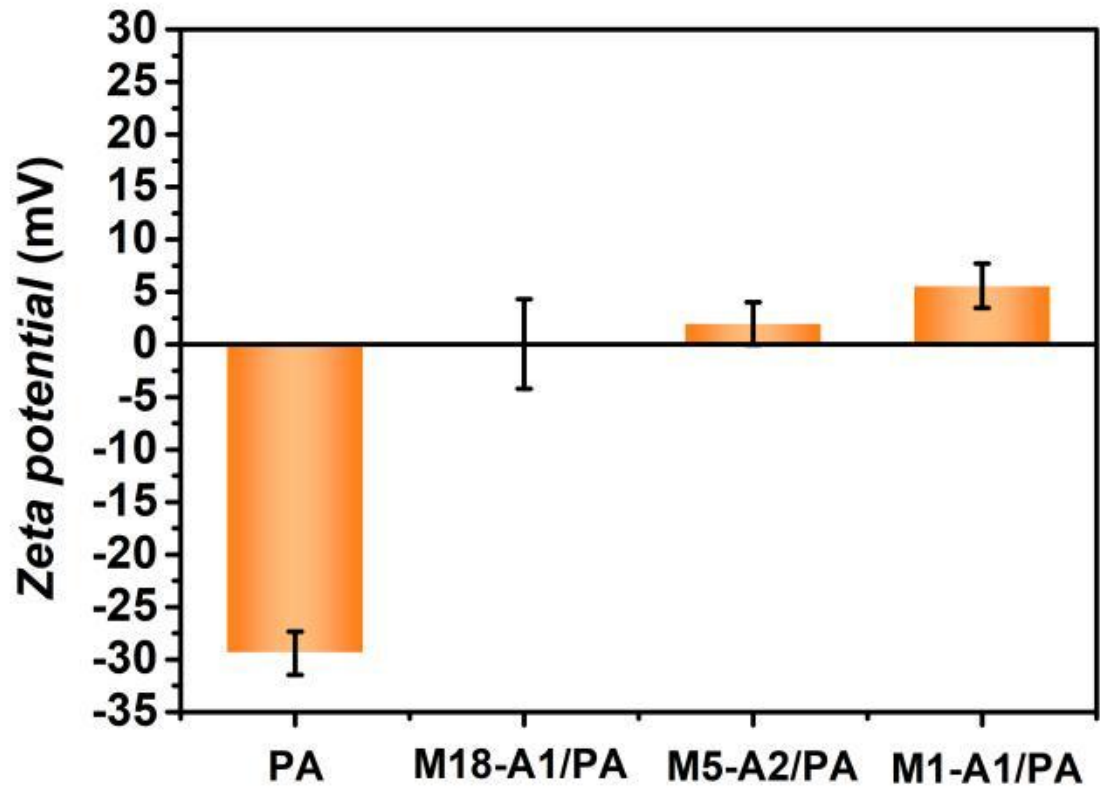

Figure S10. Zeta potential at pH 7 of different membranes. The data are shown as mean ± SD, n=3.

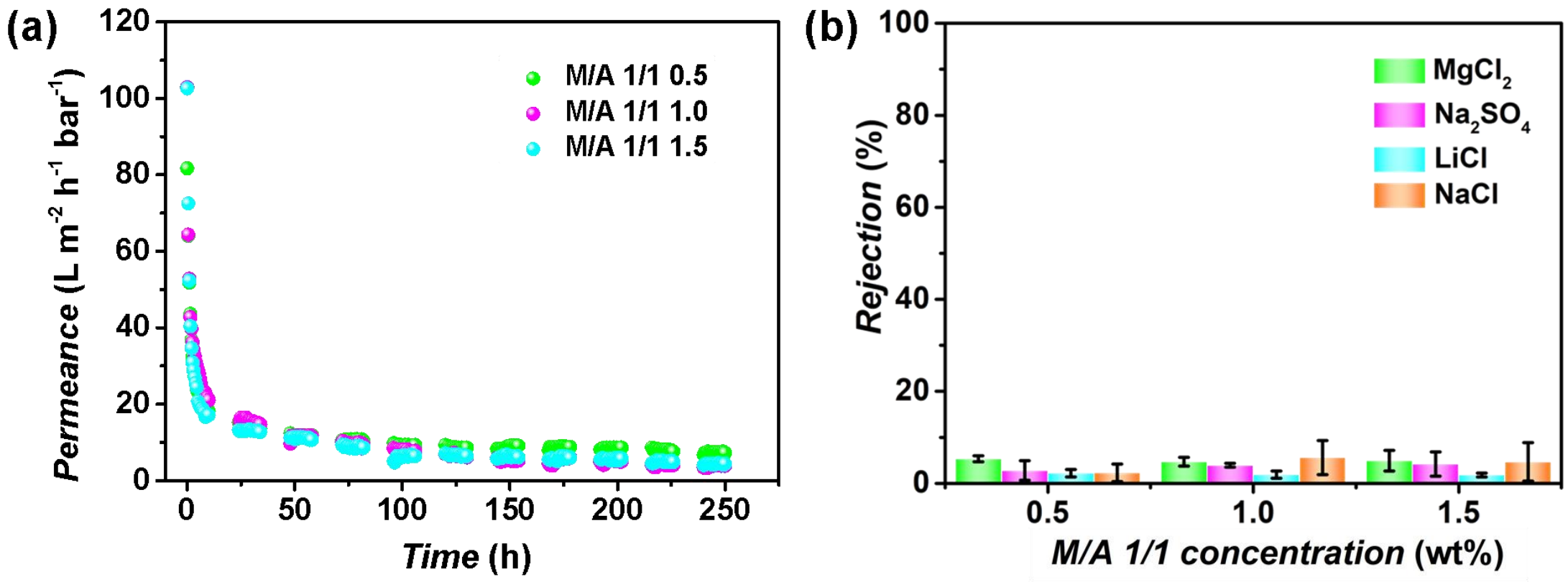

Figure S11. (a) Water permeance change over time, (b) $MgCl_2$, Na_2SO_4, LiCl, and NaCl rejections of prepared pure P(MPC–AEMA)/TMC membranes (M/A 1/1) with different concentration (0.5, 1.0, 1.5 wt%). A salt concentration of 2000 ppm was used for each single-salt feed solution. The data in b are shown as mean ± SD, n=3.

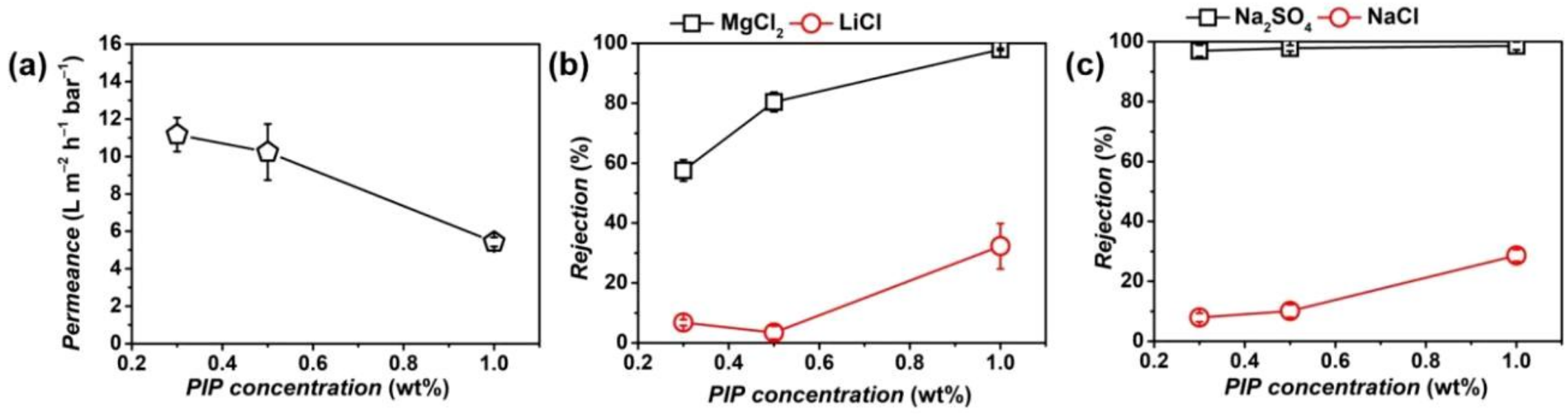

Figure S12. (a) Water permeance, (b) $MgCl_2$, LiCl, and (c) Na_2SO_4, NaCl rejections of prepared PA0.3, PA0.5, PA1.0 membrane. A salt concentration of 2000 ppm was used for each single-salt feed solution. The data are shown as mean ± SD, n=3.

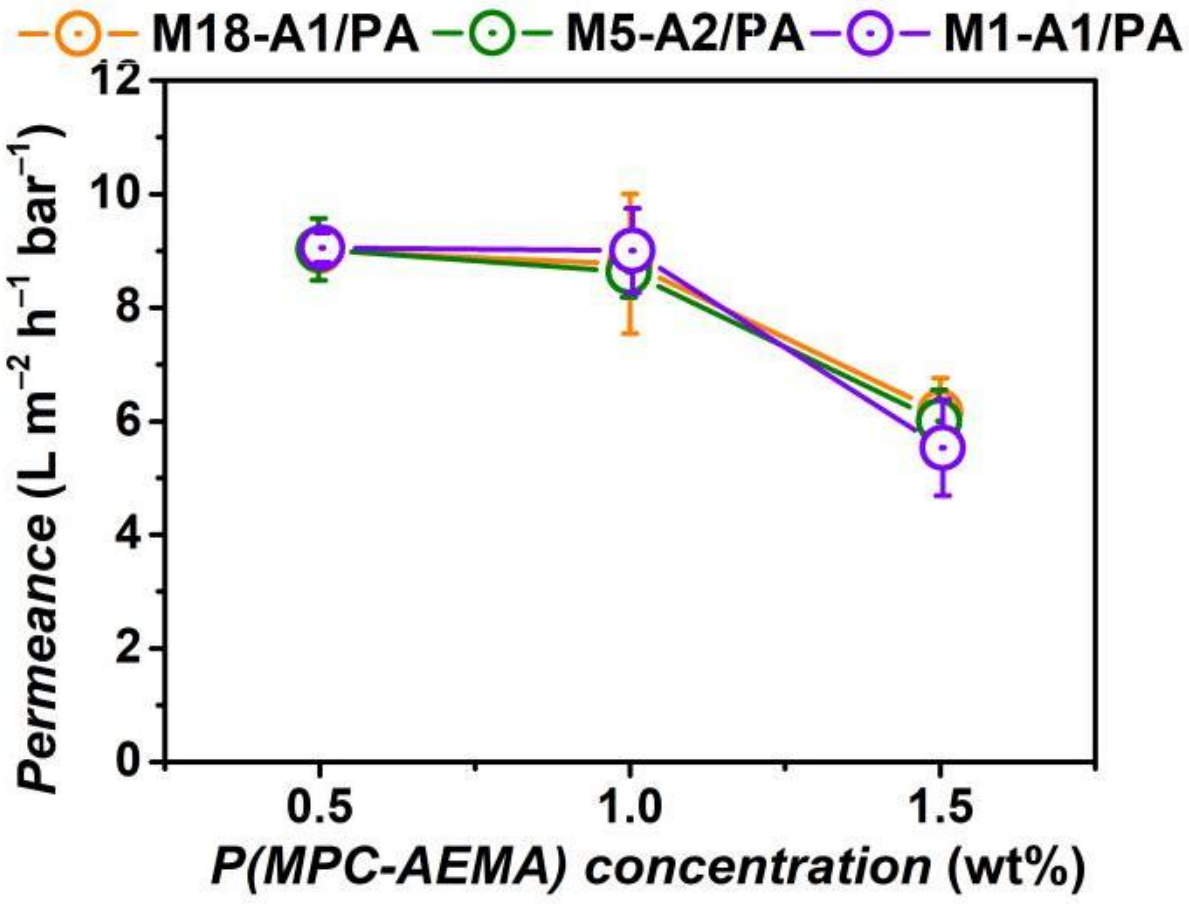

Figure S13. Water permeance of prepared M18-A1/PA, M5-A2/PA, M1-A1/PA membranes with different copolymer grafting concentrations. The data are shown as mean ± SD, n=3.

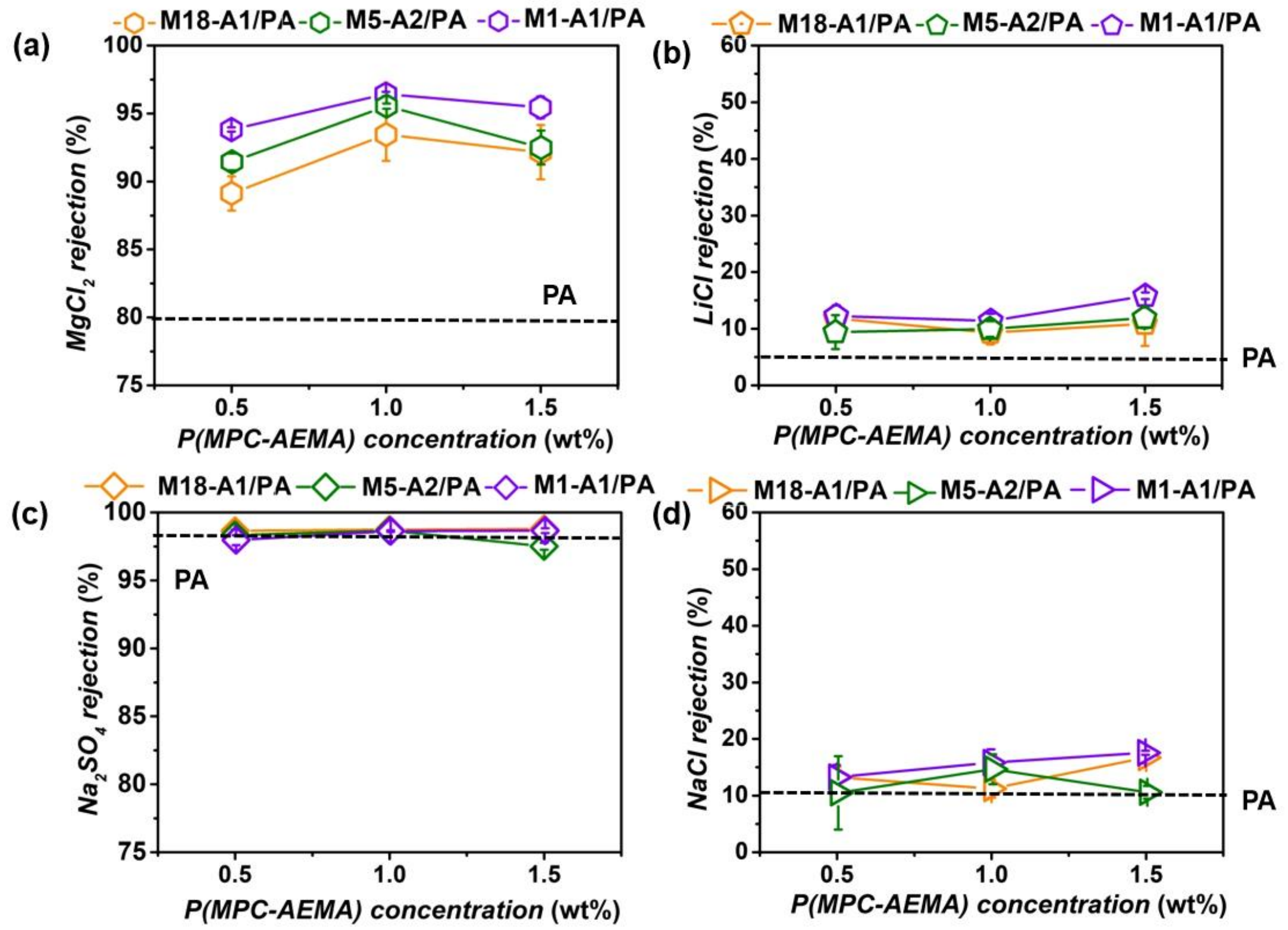

Figure S14. (a) $MgCl_2$ rejections, (b) LiCl rejections, (c) Na_2SO_4 rejections, and (d) NaCl rejections of prepared M18-A1/PA, M5-A2/PA, M1-A1/PA membranes with different copolymer grafting concentrations. A salt concentration of 2000 ppm was used for each single-salt feed solution. The data are shown as mean ± SD, n=3.

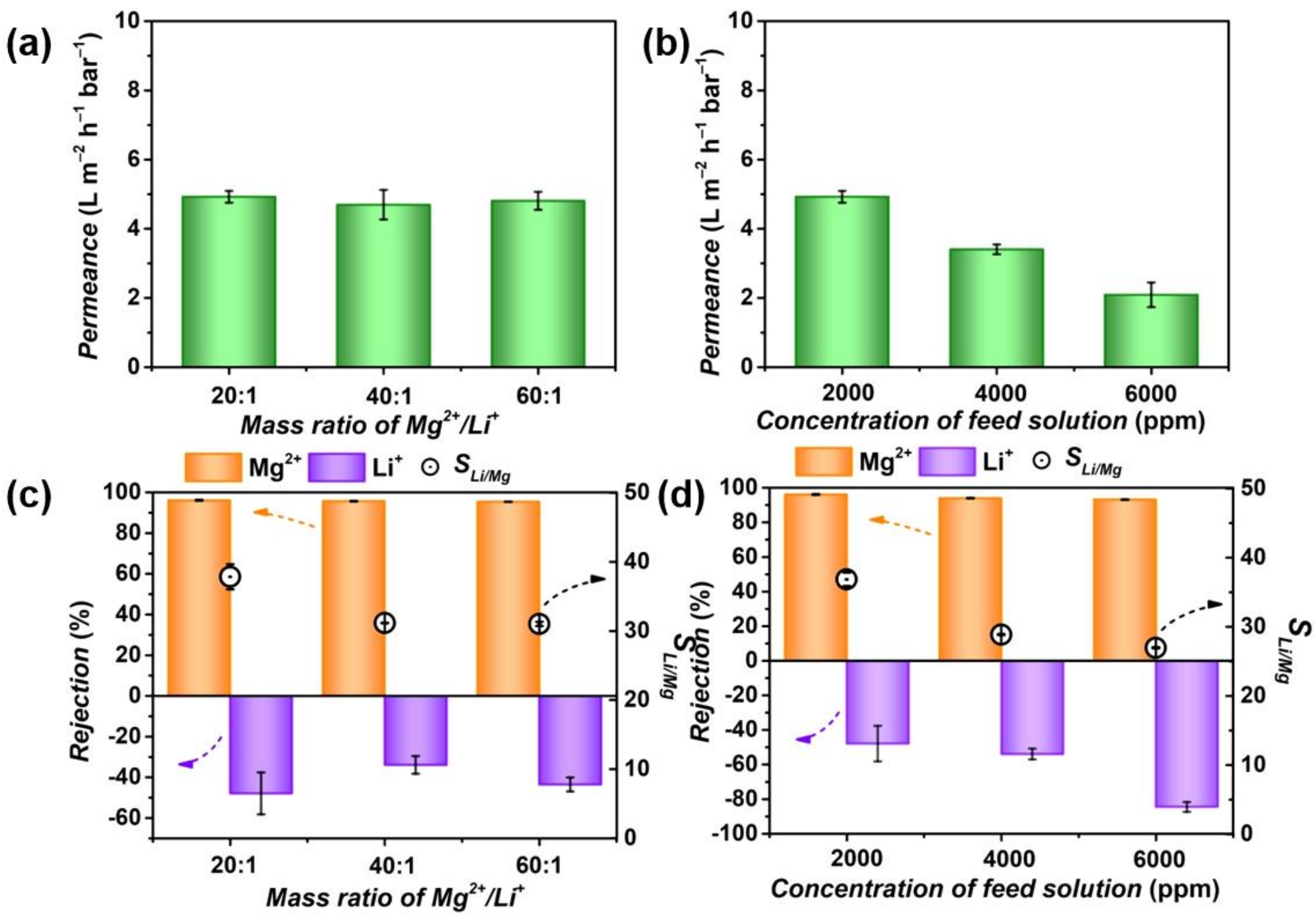

Figure S15. (a) Permeance, (c) ion rejection, and Li^+/Mg^{2+} selectivity ($S_{Li/Mg}$) of M1-A1/PA membrane by filtrating $MgCl_2$ and LiCl mixture solution with varying Mg^{2+}/Li^+ mass ratios (20, 40, 60) at a total salt concentration of 2000 ppm. (b) Permeance, (d) ion rejection, and $S_{Li/Mg}$ of M1-A1/PA membrane by filtrating $MgCl_2$ and LiCl mixture solution with different total salt concentrations (2000, 4000, 6000 ppm) at a Mg^{2+}/Li^+ mass ratio of 20. The data are shown as mean $\pm$ SD, n=3.

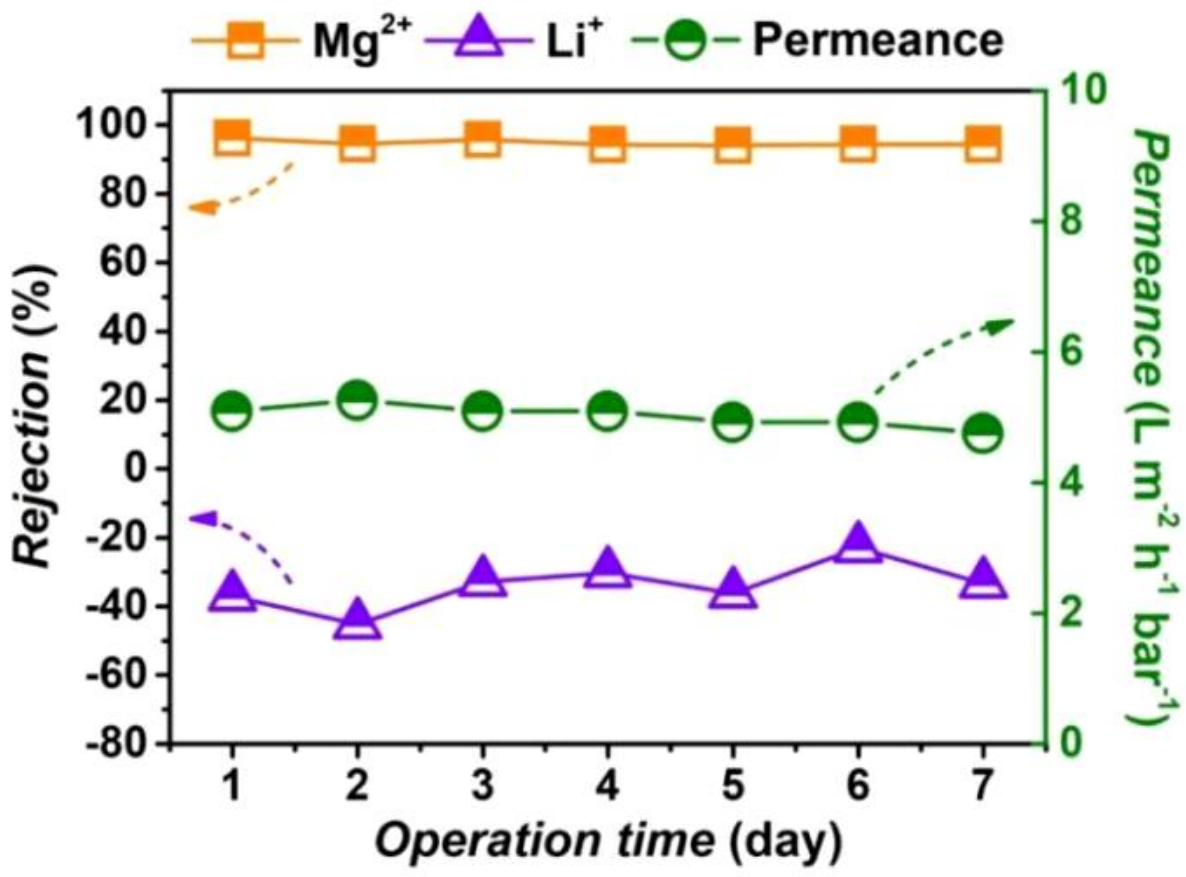

Figure S16. 7-day continuous separation test by M1-A1/PA membrane (Feed solution: 2000 ppm mixed $MgCl_2$ and LiCl solution at a Mg^{2+}/Li^{+} mass ratio of 20).

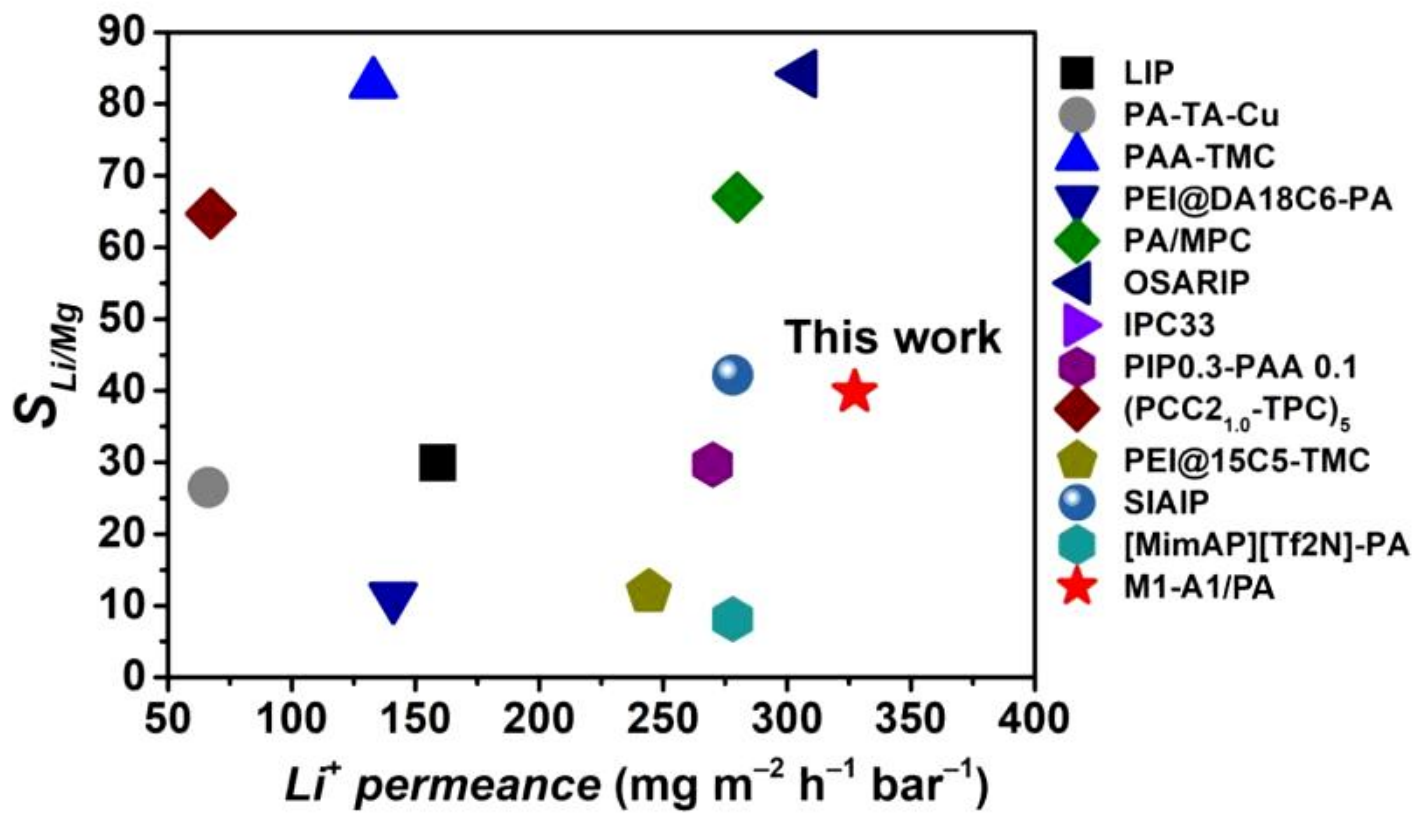

Figure S17. Comparison of Li^+ permeance and $S_{Li/Mg}$ between literature reports and M1-A1/PA membranes prepared in this work. (the details are presented in Table S3).

Table S1. The size and hydration energy of the cations and their corresponding rejections by the PA and M1-A1/PA membrane. The data are shown as mean ± SD, n=3.

	Ionic Radius (Å)	Stokes Radius (Å)	Hydration Radius (Å)	Hydration Energy (kcal mol^{-1})	Salt	PA rejection (%)	M1-A1/PA rejection (%)
Mg^{2+}	0.65	3.47	4.23	437.4	$MgCl_2$	79.0±4.45	96.1±0.24
Cu^{2+}	0.72	3.25	4.19	480.4	$CuCl_2$	79.1±0.78	92.5±0.99
Ca^{2+}	0.99	3.1	4.12	359.7	$CaCl_2$	76.8±5.69	90.4±0.16
Ba^{2+}	1.35	2.92	4.04	298.8	$BaCl_2$	72.2±5.48	92.9±0.56
Li^{+}	0.6	2.38	3.82	113.5	LiCl	5.05±2.93	11.9±0.69
Na^{+}	0.95	1.84	3.58	87.2	NaCl	7.02±1.61	14.5±2.03
K^{+}	1.33	1.25	3.31	70.5	KCl	8.66±0.83	15.1±1.34
Cs^{+}	1.69	1.19	3.29	59.8	CsCl	9.04±1.67	16.6±0.86
Rb^{+}	1.48	1.18	3.29	65.7	RbCl	7.53±2.64	16.5±1.32

Table S2. Comparison of water permeance and single-salt rejection towards NaCl, Na_2SO_4, LiCl, and $MgCl_2$ among literature reports and the membranes prepared in this work.

Membranes	Permeance (L m^{-2} h^{-1} bar^{-1})	Rejection to $MgCl_2$ (%)	Rejection to LiCl (%)	Rejection to Na_2SO_4 (%)	Rejection to NaCl (%)	$S_{LiCl/MgCl2}$	$S_{NaCl/Na2SO4}$	Ref.
LIP	10.2	95.9	21.0	~40	~30	19.3	1.17	[1]
PA-TA-Cu	4.87	95.9	17.1	~78	~35	20.2	2.95	[2]
(PES-GO)/PEI/TMC	11.2	~94	~29.5	~79	~30	11.8	3.33	[3]
PEI-TMC	5.02	94.8	34.6	81.4	36.9	12.6	3.39	[4]
PEI@DA18C6-PA	10.4	96.3	43.4	40.9	51.4	15.3	0.822	[5]
PES/(PIP-PHF)-TMC	6.70	89.9	16.3	95.6	22.6	8.29	17.6	[6]
PA/MPC	8.50	97.1	5.26	98.9	5.6	32.7	85.8	[7]
PEI-PIP (1:4)	16.0	98.5	21.0	98.8	23.5	52.7	63.8	[8]
PIP0.3-PAA 0.1	12.1	94.7	12.6	83	18	16.5	4.82	[9]
GLIP	10	98.5	22.6	61.7	36.3	51.6	1.67	[10]
MBCN/TMC	5.6	97.4	36.3	42.6	52.2	24.5	0.83	[11]
(RCC20.2-TPC)5	9.4	22.8	19.1	97	24.9	1.05	25.0	[12]
(AIP)PIP/TMC	13.6	93.9	23.5	97.3	21.6	12.5	29.3	[13]
TET/TMC	17.3	95.5	27	89.2	24.8	16	6.96	[14]
TMC/BPEI/EDTA	6	84.6	68.1	83.1	69.1	2.07	1.83	[15]
PA	11.9	80.5	4.98	98.5	11.6	4.87	57.0	This work
M18-A1/PA	10.2	94.9	8.83	98.7	11.7	18.3	65.9	This work
M5-A2/PA	9	95.7	9.11	98.6	12.5	21.1	62.0	This work
M1-A1/PA	9.35	96.7	12.0	98.7	16.1	26.3	63.1	This work

Table S3. Comparison of Li^+ separation performance between M1-A1/PA membrane and literature reports.

Membranes	Li^+ Permeance (mg m^{-2} h^{-1} bar^{-1})	Rejection to Li^+ (%)	$S_{Li/Mg}$	Ref.
LIP	159	34.0	29.9	[1]
PA-TA-Cu	66.3	42.0	26.5	[2]
PAA-TMC	133	23.3	82.8	[16]
PEI@DA18C6-PA	141	~42.0	11.2	[5]
PA/MPC	280	-40.0	67.0	[7]
OSARIP	306	-48.4	84.2	[17]
IPC33	98.3	55	117	[18]
PIP0.3-PAA 0.1	270	4.60	29.7	[9]
$(RCC2_{1.0}\text{-TPC})_5$	67.4	8	64.7	[12]
PEI@15C5-TMC	244	-30	11.9	[19]
SIAIP	278	-7.1	42.2	[20]
[MimAP][Tf2N]-PA	278	-45	8.12	[21]
M1-A1/PA	327	-49.1	39.8	This work

References

[1] S. Duan, S. Jiang, Z. Li, P. Zhang, K. Guan, P. Xu, H. Matsuyama, *Desalination* **2025**, *597*, 118393, https://doi.org/10.1016/j.desal.2024.118393.

[2] S. Fang, K. Guan, S. Zhou, Q. Song, Y. Shi, W. Fu, Z. Li, P. Xu, M. Hu, Z. Mai, P. Zhang, H. Matsuyama, *Sep. Purif. Technol*. **2024,** *581*, 117577, https://doi.org/10.1016/j.desal.2024.117577.

[3] P. Xu, J. Hong, X. Qian, Z. Xu, H. Xia, Q. Q. Ni, *Desalination* **2020,** *488*, 114522, https://doi.org/10.1016/j.desal.2020.114522.

[4] P. Xu, W. Wang, X.M. Qian, H.B. Wang, C.S. Guo, N. Li, Z.W. Xu, K.Y. Teng, Z. Wang, *Desalination* **2019,** 449, 57-68, https://doi.org/10.1016/j.desal.2018.10.019.

[5] Z. Zha, T. Li, I. Hussei, Y. Wang, S. Zhao, *J. Membr. Sci*, **2024,** *695*, 122484, https://doi.org/10.1016/j.memsci.2024.122484.

[6] Q. Shen, S. Xu, Z. Xu, H. Zhang, Z. Dong, *J. Appl. Polym. Sci.* **2019,** *136* (41), 48029, https://doi.org/10.1002/app.48029.

[7] P. Xu, S. Duan, Z. Li, M. Hu, P. Zhang, L. Dai, Z. Mai, K. Guan, and H. Matsuyama, *Adv. Funct. Mater.* **2024,** 35(10), 2416458. https://doi.org/10.1002/adfm.202416458.

[8] T. Qi, X. Chen, T. Lu, D. Jin, R. Xu, J. Zhong, *J. Membr. Sci.* **2024,** *706*, 122930, https://doi.org/10.1016/j.memsci.2024.122930.

[9] S. Duan, S. Jiang, P. Xu, Z. Li, P. Zhang, Y. Liu, A. Matsuoka, Y. Lin, J. Shen, K. Guan, T. Yoshioka, H. Matsuyama, *Adv. Sci.* **2025,** *13* (3), e13172, https://doi.org/10.1002/advs.202513172.

[10] M.-B. Wu, H. Ye, Z.-Y. Zhu, G.-T. Chen, L.-L. Ma, S.-C. Liu, L. Liu, J. Yao, Z.-K. Xu, *J. Membr. Sci.* 2022, 644, 119942. https://doi.org/10.1016/j.memsci.2021.119942.

[11] Q. Bi, C. Zhang, J. Liu, X. Liu, S. Xu, *Sep. Purif. Technol.* **2021,** *257*, 117959, https://doi.org/10.1016/j.seppur.2020.117959.

[12] Y. Zhang, Z. Hao, I. Hussein, Z. Wang, S. Zhao, *Adv. Funct. Mater*. **2024**, *34*, 2315750, https://doi.org/10.1002/adfm.202315750.

[13] G. Zhao, H. Gao, Z. Qu, H. Fan, H. Meng, *Nat. Commun*. **2023,** *14*, 7624, https://doi.org/10.1038/s41467-023-43291-2.

[14] J. Li, H. Peng, K. Liu, Q. Zhao, *Adv. Mater.* **2024,** *36*, e2309406, https://doi.org/10.1002/adma.202309406.

[15] W. Li, C. Shi, A. Zhou, X. He, Y. Sun, J. Zhang, *Sep. Purif. Technol.* **2017**, *186*, 233, https://doi.org/10.1016/j.seppur.2017.05.044.

[16] P Xu, R. R. Gonzales, J. Hong, K. Guan, Y-H. Chiao, Z. Mai, Z. Li, S. Rajabzadeh, H. Matsuyama, *J. Membr. Sci.* **2023,** *668*, 121251, https://doi.org/10.1016/j.memsci.2022.121251.

[17] Q. Peng, R. Wang, Z. Zhao, S. Lin, Y. Liu, D. Dong, Z. Wang, Y. He, Y. Zhu, J. Jin, L. Jiang, *Nat. Commun.* **2024,** *15*, 2505. https://doi.org/10.1038/s41467-024-46887-4.

[18] P. Xu, K. Guan, Y. H. Chiao, Z. Mai, Z. Li, M. Hu, P. Zhang, R. R. Gonzales, H. Matsuyama, *J. Membr. Sci.* **2023,** *688*, 122133. https://doi.org/10.1016/j.memsci.2023.122133.

[19] H. Li, Y. Wang, T. Li, X.-K. Ren, J. Wang, Z. Wang, S. Zhao, *Chem. Eng. J.* **2022,** *438*, 135658. https://doi.org/10.1016/j.cej.2022.135658.

[20] R. Jia, X.-G. Jin, Z.-L. Xu, L.-K. Wu, Y.-H. Tong, H.-X. Li, H.-H. Ping, X.-H. Ma, S.-J. Xu, *J. Membr. Sci.* **2024,** *712*, 123235, https://doi.org/10.1016/j.memsci.2024.123235.

[21] H. Wu, Y. Lin, W. Feng, T. Liu, L. Wang, H. Yao, X. Wang, *J. Membr. Sci.* **2020,** *603*, 117997, https://doi.org/10.1016/j.memsci.2020.117997.
